# Implementation strategy in collaboration with people with lived experience of mental illness to reduce stigma among primary care providers in Nepal (RESHAPE): protocol for a type 3 hybrid implementation effectiveness cluster randomized controlled trial

**DOI:** 10.1186/s13012-022-01202-x

**Published:** 2022-06-16

**Authors:** Brandon A. Kohrt, Elizabeth L. Turner, Dristy Gurung, Xueqi Wang, Mani Neupane, Nagendra P. Luitel, Muralikrishnan R. Kartha, Anubhuti Poudyal, Ritika Singh, Sauharda Rai, Phanindra Prasad Baral, Sabrina McCutchan, Petra C. Gronholm, Charlotte Hanlon, Heidi Lempp, Crick Lund, Graham Thornicroft, Kamal Gautam, Mark J. D. Jordans

**Affiliations:** 1 Division of Global Mental Health, Department of Psychiatry, George Washington University, Washington D.C., USA; 2 Department of Biostatistics and Bioinformatics and Duke Global Health Institute, Duke University, Durham, NC USA; 3 Transcultural Psychosocial Organization Nepal (TPO Nepal), Pokhara, Nepal; 4 Transcultural Psychosocial Organization Nepal (TPO Nepal), Kathmandu, Nepal; 5 King’s Health Economics, IOPPN, King’s College London, London, UK; 6 Department of Sociomedical Sciences, Columbia University, New York, NY USA; 7 Division of Global Mental Health, Department of Psychiatry, George Washington University, Washington, D.C., 20036 USA; 8 Jackson School of International Studies and Department of Global Health, University of Washington, Seattle, USA; 9 Non-communicable Disease and Mental Health Section, Epidemiology and Disease Control Division (EDCD), Department of Health Services (DoHS), Ministry of Health and Population (MoHP), Kathmandu, Nepal; 10 Duke Global Health Institute, Duke University, Durham, NC USA; 11 Centre for Global Mental Health and Centre for Implementation Science, Health Service and Population Research Department, Institute of Psychiatry, Psychology and Neuroscience, King’s College London, London, UK; 12 Centre for Global Mental Health, Health Service and Population Research Department, Institute of Psychiatry, Psychology and Neuroscience, King’s College London, London, UK; 13 Department of Psychiatry, School of Medicine and Centre for Innovative Drug Development and Therapeutic Trials for Africa (CDT-Africa), College of Health Sciences, Addis Ababa University, Addis Ababa, Ethiopia; 14 Centre for Rheumatic Diseases, Department of Inflammation Biology, School of Immunology and Microbial Sciences, Faculty of Life Sciences & Medicine, King’s College London, London, UK; 15 Alan J Flisher Centre for Public Mental Health, Department of Psychiatry and Mental Health, University of Cape Town, Cape Town, South Africa; 16 Centre for Global Mental Health and Centre for Implementation Science, Institute of Psychiatry, Psychology and Neuroscience, King’s College London, London, UK; 17 Health Service and Population Research Department, Institute of Psychiatry, Psychology and Neuroscience, Center for Global Mental Health, King’s College London, London, UK

**Keywords:** Cost-effectiveness, Developing countries, Depression, Primary care, Randomized controlled trial, Stigma, Training

## Abstract

**Background:**

There are increasing efforts for the integration of mental health services into primary care settings in low- and middle-income countries. However, commonly used approaches to train primary care providers (PCPs) may not achieve the expected outcomes for improved service delivery, as evidenced by low detection rates of mental illnesses after training. One contributor to this shortcoming is the stigma among PCPs. Implementation strategies for training PCPs that reduce stigma have the potential to improve the quality of services.

**Design:**

In Nepal, a type 3 hybrid implementation-effectiveness cluster randomized controlled trial will evaluate the implementation-as-usual training for PCPs compared to an alternative implementation strategy to train PCPs, entitled Reducing Stigma among Healthcare Providers (RESHAPE). In implementation-as-usual, PCPs are trained on the World Health Organization Mental Health Gap Action Program Intervention Guide (mhGAP-IG) with trainings conducted by mental health specialists. In RESHAPE, mhGAP-IG training includes the added component of facilitation by people with lived experience of mental illness (PWLE) and their caregivers using PhotoVoice, as well as aspirational figures. The duration of PCP training is the same in both arms. Co-primary outcomes of the study are stigma among PCPs, as measured with the Social Distance Scale at 6 months post-training, and reach, a domain from the RE-AIM implementation science framework. Reach is operationalized as the accuracy of detection of mental illness in primary care facilities and will be determined by psychiatrists at 3 months after PCPs diagnose the patients. Stigma will be evaluated as a mediator of reach. Cost-effectiveness and other RE-AIM outcomes will be assessed. Twenty-four municipalities, the unit of clustering, will be randomized to either mhGAP-IG implementation-as-usual or RESHAPE arms, with approximately 76 health facilities and 216 PCPs divided equally between arms. An estimated 1100 patients will be enrolled for the evaluation of accurate diagnosis of depression, generalized anxiety disorder, psychosis, or alcohol use disorder. Masking will include PCPs, patients, and psychiatrists.

**Discussion:**

This study will advance the knowledge of stigma reduction for training PCPs in partnership with PWLE. This collaborative approach to training has the potential to improve diagnostic competencies. If successful, this implementation strategy could be scaled up throughout low-resource settings to reduce the global treatment gap for mental illness.

**Trial registration:**

ClinicalTrials.gov, NCT04282915. Date of registration: February 25, 2020.

**Supplementary Information:**

The online version contains supplementary material available at 10.1186/s13012-022-01202-x.

Contributions to the literature

- Stigma related to mental illness among primary care providers is a barrier to accurate detection of patients with mental illnesses because providers who stigmatize do not ask about mental illness, do not conduct thorough assessments, and do not develop diagnostic competency.
- Based on the evidence for social contact interventions, a collaboration of people with lived experience of mental illness, who participate as co-facilitators in trainings for primary care providers, has the potential to reduce stigma as well as improve the reach of services through accurate detection of mental illnesses.
- A type 3 hybrid implementation-effectiveness cluster randomized controlled trial in Nepal will evaluate the stigma and detection outcomes comparing implementation-as-usual training, which is based on the World Health Organization mental health Gap Action Programme (mhGAP), vs. an alternative implementation strategy integrating people with lived experience of mental illness into mhGAP training.

## Introduction

### Background and rationale

There continues to be a major gap globally between the number of people living with mental illnesses and the number of people receiving minimally adequate treatment. In the USA and other high-income countries, approximately 1 out of 5 persons receive minimally adequate care for depression [1]. In low- and middle-income countries (LMICs), the number of people receiving minimally adequate care ranges from 1 out of 27 to 1 out of 100 for conditions including depression, anxiety, and substance use disorders [1–3] and 1 out of 6 for psychosis [4]. To address this gap in LMICs, a key strategy has been training primary care providers (PCPs) in the diagnosis and treatment of people with mental illnesses. The World Health Organization (WHO) developed the mental health Gap Action Programme Intervention Guide (mhGAP-IG) to train PCPs to detect people with mental illnesses and deliver evidence-supported interventions [5].

However, research to date suggests that implementation strategies for mhGAP-IG and similar initiatives are not yielding the optimal benefit of these primary care-based strategies. In a recent meta-analysis covering 12 LMICs, the pooled depression detection rate in primary care was 7% [6]. In Kenya, only 5% of primary care facilities detected one or more people with mental illnesses 3 months after training [7]. In Ethiopia, only 1.3% of patients with depression were accurately detected by mhGAP-trained primary care workers [8]. In Malawi, only 1 out of 10 patients with depression and 1 out of 100 patients with anxiety were correctly identified by trained PCPs [9]. In Nepal, fewer than half of patients with mental illnesses were correctly identified by the mhGAP-trained staff in primary care facilities, and for depression, only 1 out of 5 were correctly identified [10]. Similarly, in Nepal, psychosis was also accurately diagnosed among fewer than 1 out 10 patients [11].

Despite poor identification of mental illness, studies of primary care services in LMICs, including in Nepal, demonstrate that the correct treatments are provided if a patient is accurately diagnosed, and this leads to improved patient outcomes [10, 12, 13]. This suggests that the main bottleneck in expanding primary care services is at the level of *detection*. The treatments are effective, but they are not adequately reaching the appropriate people, i.e., the right people are not given the right diagnosis to initiate the right treatment in a timely manner.

Stigma among PCPs against people with mental illness has been identified as a contributor to low detection rates in primary care-based mental health services [7–9, 14–21]. This is because PCPs who stigmatize do not ask about mental illnesses, do not conduct thorough assessments, and do not develop diagnostic competency [15, 16, 22–25]. Therefore, one avenue to improve the accurate detection of mental illnesses among patients in primary care is to integrate stigma reduction when training PCPs. In a review of more than 162 studies of mhGAP-IG trainings, only 15 had completed evaluations of stigma, of which 9 (60%) reported a reduction in stigma, and most of these had an extra explicit anti-stigma component added to standard mhGAP-IG curricula; none were randomized controlled trials (RCTs) [13]. There is research from fields outside of mental health in which other stigmatized conditions, such as HIV/AIDS, have shown an association between stigma reduction and improved clinical detection and care [26–30].

### Preliminary studies of the RESHAPE implementation strategy

To address this research gap in stigma reduction when training PCPs in mental healthcare, we designed an intervention to support the engagement of people with lived experience of mental illness (PWLE) in the delivery of mhGAP-IG training [31]. This implementation strategy is entitled Reducing Stigma Among Healthcare Providers (RESHAPE). The conceptual foundation of RESHAPE is promoting empathy between PCPs and PWLE using intergroup contact theory from social psychology, as well as reduction of intergroup discrimination by lowering threat and anxiety, as informed by social neuroscience; this is framed as a “what matters most” approach to understand stigma using a moral framework from medical anthropology [32, 33].

The underlying tenet of RESHAPE is that when health workers feel low levels of threat (e.g., minimal risk to their own health and safety), feel professionally competent to treat people with mental illnesses, and do not feel at risk of ostracization by coworkers and community members, there will be increased empathy and willingness to initiate and follow-up care [34].

To implement RESHAPE, PWLE who are in different states of recovery and their caregivers are selected from the local community. They receive PhotoVoice training—a participatory action research method [35] that has been used to address mental illness stigma [36]—to develop recovery testimonials that are delivered in-person with personalized photographs at mhGAP-IG trainings. In addition, the PWLE are trained in public speaking and to participate in question and answer (Q&A) sessions with PCPs.

Another component of RESHAPE is the use of aspirational figures, who have previously been trained in mental healthcare and have shown high levels of motivation to treat patients with mental illness at their primary care facilities. The expectation is that PCPs in training will aspire to be like these colleagues who serve as role models. Aspirational figures are trained in myth-busting, which is a discussion of common myths and facts related to mental illness, and myth-busting has been identified as one of the active ingredients of effective stigma reduction [37]. Aspirational figures also present recovery stories of their patients. Full details on the RESHAPE strategy have been published previously [32].

Proof of concept testing of RESHAPE was conducted in Nepal [32], and a pilot cluster randomized controlled trial (cRCT) was conducted with 34 health facilities and 88 PCPs who had prescribing privileges in their health facilities [11]. Diagnostic accuracy was assessed with 69 patients. Mixed methods evaluation of the pilot cRCT demonstrated feasibility and acceptability of the RESHAPE implementation strategy. Qualitative narratives demonstrated that the PCPs felt they better understood the experience of patients and felt more confident that they could diagnose and treat such patients after hearing the recovery narratives [32, 38–41]. They also had more willingness to initiate psychological services [41].

Quantitative analyses showed that PCPs in the RESHAPE arm had a 10.6 point reduction on the Social Distance Scale (SDS) [42] compared to only a 2.8 point reduction among PCPs in the implementation-as-usual (IAU) arm [11]. In addition, PCPs in the RESHAPE arm had 72.5% accuracy in patient diagnosis compared to 34.5% accuracy among PCPs in the IAU arm [11]. In the IAU arm, depression was only accurately diagnosed in 50% of patients, and psychosis was only accurate among 7% of patients, whereas diagnoses were more accurate in the RESHAPE arm in the pilot. There were no adverse events in either arm.

All criteria for proceeding to a full trial, as specified in the pilot, were met. Based on these pilot results, which were not powered for hypothesis testing, it was warranted to proceed to a full-scale trial that would be powered for hypothesis testing for stigma reduction and improved accuracy of detection.

### Objectives

The cRCT has two co-primary objectives. Primary objective 1 is to determine the effect of the RESHAPE implementation strategy on stigma among PCPs (see Additional file 1: Fig. S1). This objective evaluates the attitudinal change among PCPs. *Hypothesis:* PCPs in the RESHAPE arm will have a greater reduction in stigma toward people with mental illness 6 months after training compared with primary care workers exposed to the standard training in IAU.

Primary objective 2 is to evaluate the effect of the RESHAPE implementation on reach. In the Reach Effectiveness-Adoption Implementation Maintenance (RE-AIM) framework [43–45], “*reach* is the number, proportion of the intended audience, and the representativeness of participants compared with the intended audience” [46]. Reach will be operationalized as the accuracy of diagnosis among PCPs. *Hypothesis*: PCPs in the RESHAPE arm will have greater reach in terms of a greater proportion of patients accurately diagnosed compared to IAU.

Primary objective 2 is a type 3 *implementation-effectiveness* objective because the implementation outcome (reach) is the primary focus. The effectiveness component, which is secondary, addresses an intervention at the patient level that is the same in both arms (i.e., mhGAP-IG recommended pharmacological and psychological treatments, see Table 1). It is only the implementation strategy with regard to how PCPs are trained which differs between the arms (implementing training-as-usual vs. RESHAPE). Therefore, one of the secondary objectives relates to the effectiveness of the care delivered. We hypothesize that RESHAPE will have non-inferior outcomes compared to IAU, i.e., a non-inferiority hypothesis. We have selected a non-inferiority approach because prior research in Nepal showed that, 6 months after training, PCPs delivered minimally adequate care for 94% of patients with depression and 95% of patients with alcohol use disorder [10]. Therefore, it is unlikely to improve upon this outcome when using RESHAPE, we thus want to test if the treatment outcomes are comparable across arms. This echoes the point raised above that detecting who needs care appears to be a bigger challenge than providing the right care once appropriate persons are identified.

**Table 1:** Intervention and implementation elements for implementation as usual (IAU) vs. RESHAPE

	Implementation as usual (IAU)	RESHAPE implementation
*mhGAP (**www.who.int/mental_health/mhGAP/training_manuals**)*		
Diagnosis of depression, generalized anxiety disorder, psychosis, and alcohol use disorder	X	X
Pharmacological treatment of these conditions	X	X
Psychosocial treatment of these conditions	X	X
*RESHAPE PhotoVoice and associated training of PWLE, caregivers, and aspirational figure*		
Recruitment and PhotoVoice training of PWLE and caregivers		X
Recruitment and training of aspirational figures		X
*mhGAP and associated training of primary care providers*		
mhGAP training on diagnosis delivered by a psychiatrist	X	X
mhGAP training on medication management delivered by a psychiatrist	X	X
Psychosocial training by a MPhil psychologist	X	X
PWLE and caregiver recovery stories and Q&A		X
Aspirational figures describe experiences of providing mental health care and Q&A		X
Collaborative brain-storming with aspirational figures		X

*Abbreviations*: *mhGAP* mental health Gap Action Programme, *PWLE* people with lived experience of mental illness, *RESHAPE* Reducing Stigma Among Healthcare Providers

Another secondary objective is to evaluate stigma reduction as a potential mediator of differences in reach (see Additional file 1: Fig. S1). By evaluating stigma as a mediator of reach, this can determine what degree of stigma change may be clinically relevant in terms of accurately diagnosing patients. The contribution of stigma to reach can be evaluated in multi-mediation models that also take factors such as knowledge and competency into account. Evaluation of the cost-effectiveness of the RESHAPE strategy is also a secondary objective. Additional file 1: Fig. S2 provides the full list of outcomes using the RE-AIM framework.

## Methods

### Trial design

**Fig. 1:**
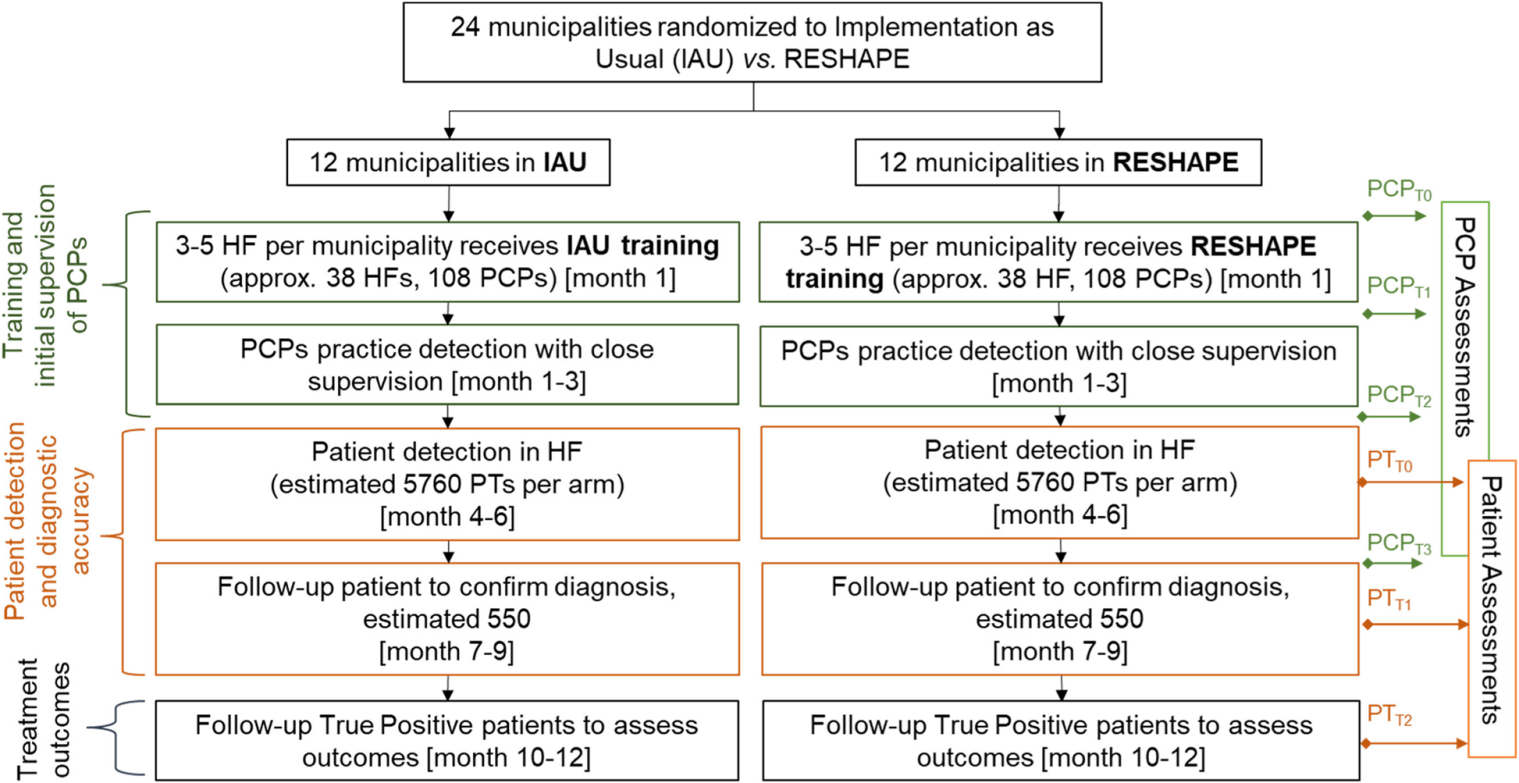
Cluster randomized controlled trial CONSORT flow chart. Abbreviations: HF, health facility; PCP, primary care provider; PT, patient; RESHAPE, Reducing Stigma Among Healthcare Providers

The study design will be a parallel two-arm cRCT in Nepal randomizing 24 municipalities (i.e., clusters) in a 1:1 ratio to one of two different implementation strategies (see Fig. 1). The two arms are the Nepali government version of mhGAP taught by mental health specialists vs. the *RESHAPE* arm, which is the Nepali mhGAP taught by specialists and PWLE, their caregivers, and aspirational figures. Given the focus of this research on real-world implementation, intention-to-treat is our primary framework.

A cluster design was selected because of the potential contamination of implementation strategies among PCPs at the same facility, and the likelihood of shared patient management among PCPs. One change from the pilot cRCT to the current study was changing the level of clustering from the health facility (pilot design) to municipality (current full-scale design). This is because of the risk of government re-assignment of PCPs from one facility to another in the same municipality. Also, because of high rates of turnover, there is a risk of losing entire clusters if all PCPs leave. Therefore, by having multiple health facilities in a cluster, there was less risk of cluster loss.

Three to five health facilities per municipality will be enrolled: approximately 38 per arm, which equates to 76 facilities for the entire study. Approximately 1–3 PCPs will be enrolled per health facility, with a target of 108 PCPs per arm. All health facilities within a municipality and all PCPs in those health facilities will be in the same arm, i.e., there is no mixing of arms within facilities or within municipalities. Across both arms, the goal will be to enroll approximately 1100 total patients in the trial for diagnostic evaluations of depression, generalized anxiety disorder, psychosis, or alcohol use disorder. The study will report results for total accuracy and is powered for total patient accuracy. In addition, outcomes per mental health condition will be reported.

### Study setting

Nepal was selected as the site for this study because of our extensive preliminary research in this setting and because it exemplifies a low-resource context where the treatment gap for mental illness is high [47]. In a country of 29 million people, Nepal has approximately 200 psychiatrists, i.e., 1.45 psychiatrists per 100,000 population [48], compared to 8 psychiatrists per 100,000 population in the USA. Nepal has high exposure to negative social determinants of health (war, environmental disasters, poverty, and gender-based and ethnic discrimination [49–66]), and the government is the main provider of healthcare throughout the country. Depression rates vary from 10 to 40% based on the setting [54, 60, 61, 67–71], with prevalence at primary care facilities of approximately 17% based on Nepali-validated Patient Health Questionnaire (PHQ-9) scoring [64]. Suicide was recently the leading single cause of mortality among women of reproductive age [72]. In rural Nepal, 90% of female suicides occur before 25 years of age [59]. Among patients attending primary care, 11% report suicidal ideation and 1.2% attempted suicide in the past year [60]. There is also increasing political will to address mental health: the Nepal Ministry of Health and Population endorsed the National Mental Health Strategy and Action Plan in 2021 [73]. Please see Additional file 1 for additional information on the *study setting and healthcare workforce in Nepal*.

### Interventions

In keeping with hybrid implementation-effectiveness trials, an evidence-supported intervention is needed as the basis of which to evaluate different implementation strategies; this will be the mhGAP-IG (www.who.int/mental_health/mhgap) for our study [74, 75]. The mhGAP-IG is the standard for primary care-based mental health services in LMIC and is being implemented in more than 100 countries [74]. Materials for mhGAP have been translated and adapted for Nepal and evaluated through the Programme for Improving Mental health Care (PRIME) [76]. The mhGAP package, developed and validated with demonstrated evidence in Nepal, was a 9-day training on 4 mhGAP mental health conditions (depression, psychosis, alcohol use disorder, and epilepsy). The government has modified this to a 6-day training with 10 mental health conditions (depression, anxiety, psychosis, alcohol use disorder, epilepsy, conversion disorder, suicide, dementia, child and adolescent mental and behavioral disorders, and other significant mental health complaints). Of note, WHO mhGAP-IG does not include anxiety as a diagnosis. However, the Nepal government added generalized anxiety disorder to their national mhGAP. There was no involvement of PWLE in the decision of the government to transition from the evidence-based PRIME version of the mhGAP curriculum to the established government curriculum. This trial will use the government-approved curriculum. The conditions of interest for evaluation in this study, out of the 10 covered mental health conditions, will be depression, generalized anxiety disorder, psychosis, and alcohol use disorder.

Trainings are led by 1–2 Nepali psychiatrists who have previously participated in a training-of-trainers program to learn how to teach mhGAP-IG. An MPhil level psychologist teaches the psychosocial components. For each mental health condition, the psychiatrist introduces the hallmark symptoms, discusses medication management and psychosocial interventions, and lists considerations for diagnosis and treatment with special populations, e.g., during pregnancy or when the person has a co-morbid medical condition. Pharmacological regimens for each mental health condition have been adapted according to what medications are freely available in Nepal.

**Fig. 2:**
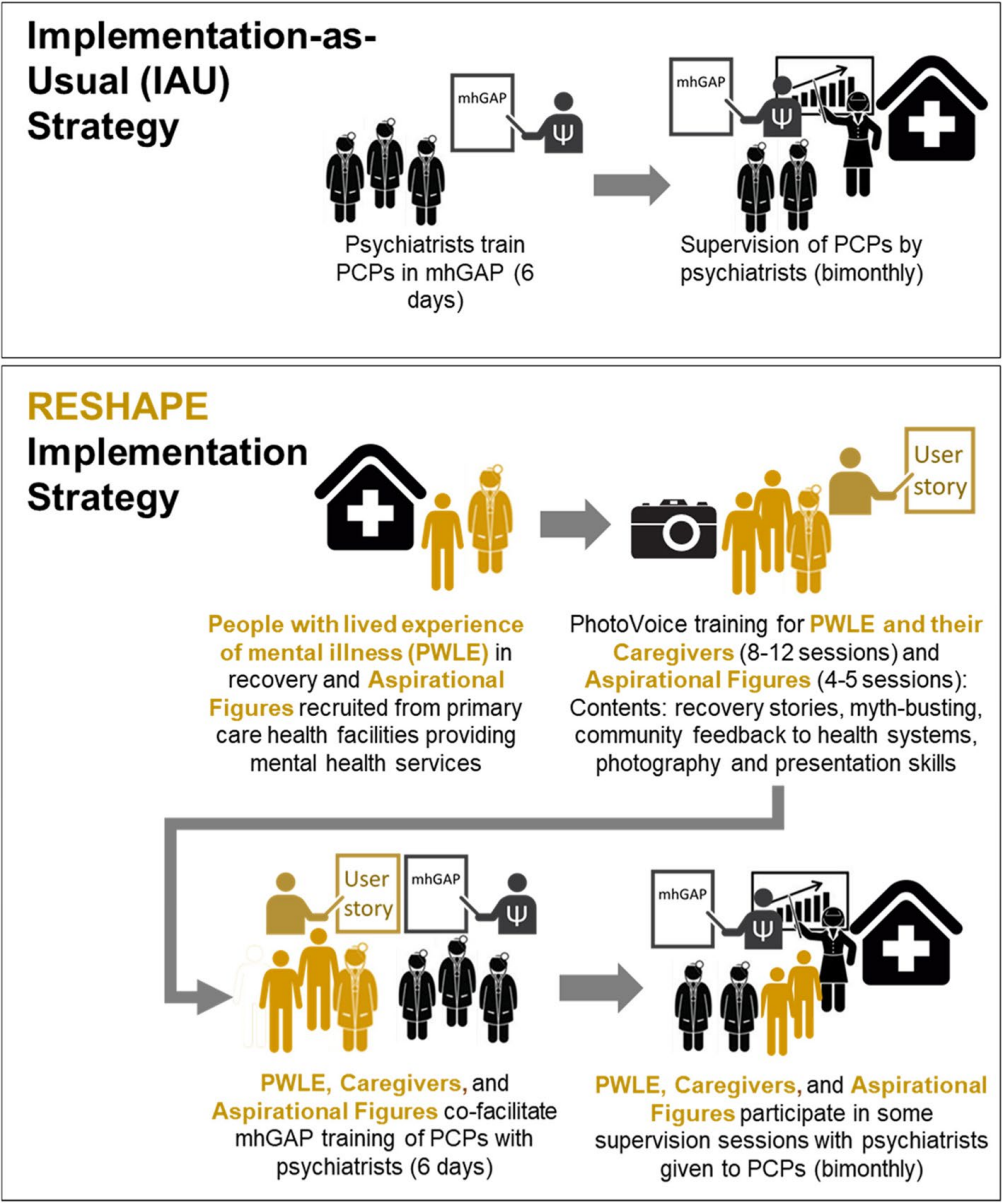
Procedures for implementation as usual vs. RESHAPE. Abbreviations: mhGAP, mental health Gap Action Programme; PCP, primary care provider; RESHAPE, Reducing Stigma Among Healthcare Providers

In the RESHAPE implementation arm, the mhGAP-IG training lasts the same duration (6 days) and covers the same content; however, the teaching style is different (see Fig. 2). For RESHAPE, instead of each mental health condition only being taught by a psychiatrist, there is also a PWLE and potentially his/her caregiver who will present a recovery narrative about living with that particular mental health condition. PWLE and their caregivers also participate in brief Q&A sessions where PCPs can ask them about living with the condition, their treatment, and other topics of interest. There is typically one PWLE and potentially his/her caregiver taking part in each session for the key mental health conditions, i.e., one PWLE of depression participating in the mhGAP-IG depression module section, a PWLE of generalized anxiety disorder, a PWLE of psychosis, and a PWLE of alcohol use disorder. Thus, there are approximately 4 PWLE sharing recovery narratives during the training. In addition, videos may be used to supplement some modules, e.g., a video of a PWLE of depression [77].

In addition, the aspirational figures participate in two sessions. One session is on day 2 about myths and facts related to mental illness, and the other session is on day 5 to discuss anticipated challenges and barriers when implementing mental health services in primary care. An aspirational figure may also accompany a PWLE, such as for the psychosis recovery narrative.

The RESHAPE implementation strategy is time-matched with IAU training. For example, in IAU, the psychiatrist will spend 2 h describing the diagnosis and treatment of depression whereas in RESHAPE, the psychiatrist would spend 90 min in didactic training followed by a 30-min presentation by a PWLE and Q&A. This means that in RESHAPE, the PCPs in training receive less time with didactics exclusively taught by a psychiatrist.

In order to prepare PWLE and their caregivers for participation in mhGAP-IG trainings, PWLE participate in approximately 8–12 sessions of PhotoVoice training [39] to develop their recovery narrative, practice public speaking skills, and learn distress management skills in case of any emotional distress experienced while participating in the training. The aspirational figures participate in approximately 4–5 sessions of training to practice myth-busting and to prepare narratives about their experiences of delivering mental health services. Full details of PWLE, caregiver, and aspirational figure training are provided elsewhere [32].

### Outcomes

**Fig. 3:**
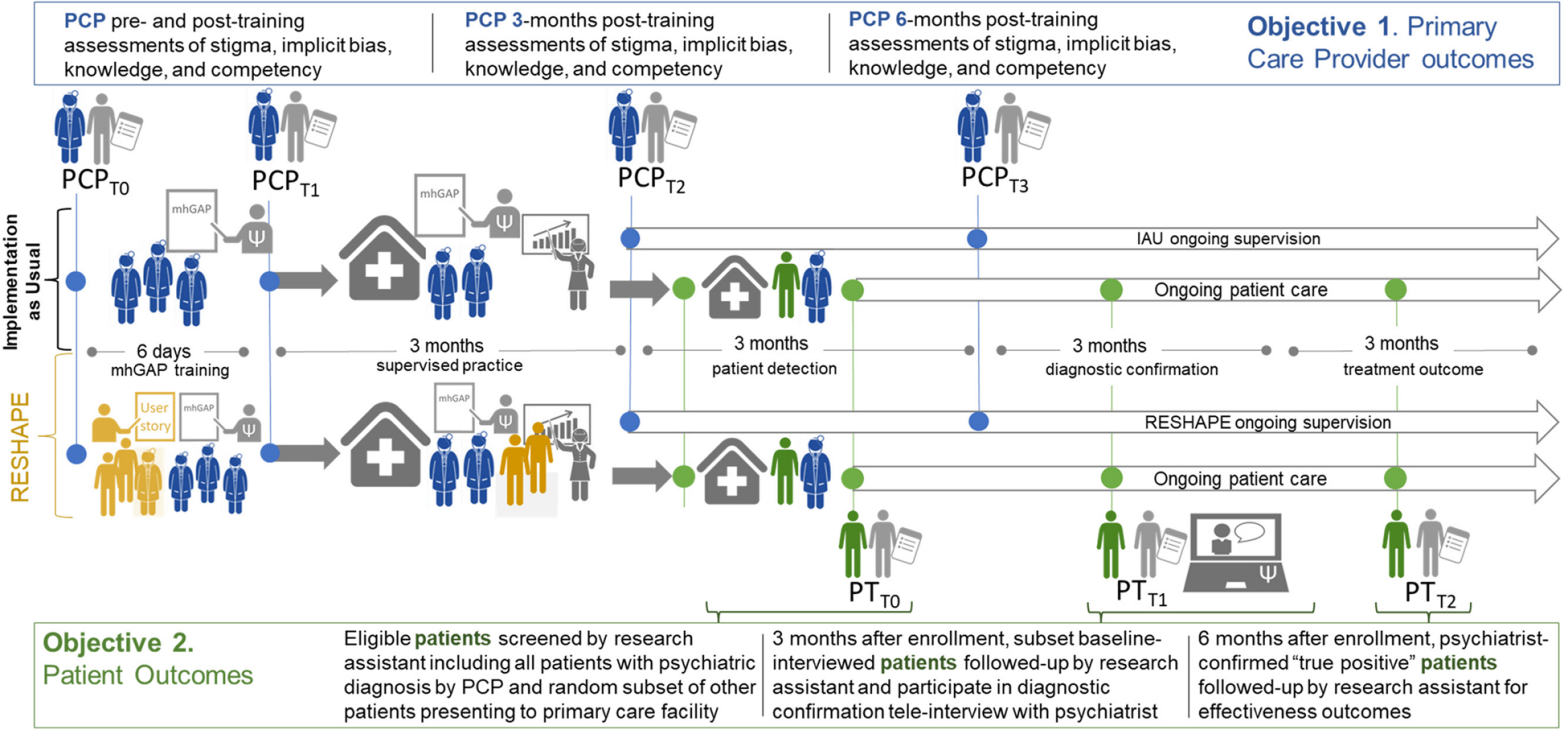
Data collection pathway for primary care providers (PCPs) and patients (PT) in primary care facilities. Abbreviations: mhGAP, mental health Gap Action Programme; RESHAPE, Reducing Stigma Among Healthcare Providers; IAU, implementation as usual

PCP outcomes will include primary objective 1 and a number of secondary outcomes (Table 2 and Fig. 3). The primary outcome is the Social Distance Scale (SDS), which is a commonly used measure of stigma [42, 78, 79]. The SDS was developed in the 1920s by Bogardus [42] to measure the level of acceptability of various types of social relationships between Americans and members of common ethnic groups [78, 112]. The modified SDS has been widely used to measure mental health-related stigma [78, 113]. The SDS measures the acceptability of different degrees of social distance and thus, by inference, the attitude of the respondent to the person with the condition [114]. A commonly used version consists of questions that represent social contact with different degrees of distance, such as renting a room to someone with a condition under study, working in the same place, marrying one’s child to a person with the conditions, or engaging someone in child care. The SDS sum score represents the attitude of the respondent toward the condition. The SDS has been adapted for use both with and without vignettes, using a 6-point scale and 12 items. Cross-cultural use of SDS in LMIC across health conditions has recently been reviewed [115]. In this trial, PCPs will be presented with 3 versions of the SDS, in random order. Each version includes a vignette followed by 12 questions regarding willingness to engage with the person in the vignette. These 12 questions have been culturally adapted for Nepal and other LMICs [116]. The three vignettes represent persons with depression, psychosis, and alcohol use disorder. Of note, an SDS vignette for generalized anxiety disorder was not added in addition to depression, given that the current three vignettes cover common mental health conditions, severe mental health conditions, and substance use conditions. Moreover, it would add to respondent burden in terms of additional questions and may lead to respondent inattention given the repetition of the same questions multiple times.

**Table 2:** Study measures

Domain	Tool
**Primary care providers (PCPs)**	
Attitudes	*Social Distance Scale (SDS)*^A, C^: explicit stigmatizing attitudes questionnaire [42] widely used in mental health [78] in global stigma comparisons [79]; the Nepali version has 12 self-report questions on a scale of 1 to 6 and three vignettes (depression, psychosis, and alcohol use disorder); Nepal *α* = 0.90. ***Primary outcome for objective 1; mediator for objective 2***. *PCP*_*T0,T1,T2,T3*_.
	*Implicit Association Test (IAT)*^B^: implicit biases against mental illness; tablet-administered test: mental health version has been used in numerous high-resource settings [80–83]; in Nepal, we developed two versions: mental illness vs. harmfulness and mental illness vs. burdensomeness; administration time is 15 min. *PCP*_*T0,T1,T2,T3*_.
Behavioral intentions	*Reported and Intended Behavior Scale (RIBS)*^B^: 8-item scale to measure the behavioral intentions towards people with mental health problems [84]. *PCP*_*T0,T1,T2,T3*_.
Knowledge	*mhGAP Knowledge*^B, C^: 31 multiple-choice questions for diagnostic and treatment knowledge; globally used for mhGAP trainings [85–87]. *PCP*_*T0,T1,T2,T3*_.
Competency and quality	*Enhancing Assessment of Common Therapeutic factors (ENACT)*^B, C^: observed structured clinical exam; health workers conduct a 10-min role-play with standardized patients; 15 items for common factors, 5 items for mhGAP assessment competencies, and 2 items for recommended diagnosis and treatment; developed in Nepal [88, 89]; *α* = 0.89; administered through the World Health Organization Ensuring Quality in Psychological Support (EQUIP) platform [90]. *PCP*_*T0,T1,T2,T3*_.
Self-efficacy	*mhGAP Clinical Self-Efficacy*^B^: 38 self-reported ability to diagnose and treat mental illness; standard mhGAP assessment; widely used globally [85–87]; Nepal *α* = 0.99. *PCP*_*T0,T1,T2,T3*_.
**Patient**	
Accurate diagnosis	*Structured Clinical Interview for DSM-5-Clinical Trials Version (SCID-5-CV)*^A^: psychiatrist interview for diagnostic accuracy [91]; SCID for DSM-IV previously used in Nepal showing strong concordance with Composite International Diagnostic Interview [92]; accurate diagnosis is used as a proxy for reach of services. ***Primary (implementation) outcome for objective 2***. *PT*_*T1*_.
Functioning	*WHO Disability Assessment Schedule (WHODAS)*^B^: self or caregiver report of functional impairment with 13 fixed response questions and 3 open-ended related timing [93]; widely used in Nepal [61, 70, 94–96]; Nepal *α* = 0.87. *PT*_*T0,T1,T2*_.
Quality of life	*EQ-5D-5L*^B^: 5-item self or caregiver report of quality of life years (QALYs) based on 5 dimensions: mobility, self-care, daily activities, pain/discomfort, and mood (anxiety/depression); official EuroQol translation available for Nepal; Asian utility weights available from Thailand [97]. *PT*_*T0,T1,T2*_.
Psychiatric symptom severity	*Patient Health Questionnaire (PHQ9)*^B^: 9-item assessment of depression symptoms and 1-item impact on functioning [98]; validated in a primary care setting in Nepal, with the addition of a local idiom of distress “heart-mind problems” [67]; validated Nepal cutoff ≥ 10: sensitivity = 94%, specificity = 80%; *α* = 0.84. *PT*_*T0,T1,T2*_.
	*Generalized Anxiety Disorder (GAD-7)*^B^: 7-item assessment of anxiety symptoms, structured in the same format as the PHQ-9. Previously used in Nepal [99–101]. *PT*_*T0,T1,T2*_.
	*Positive and Negative Syndrome Scale (PANSS)*^B^: assessment of positive and negative symptoms of psychosis, designed for schizophrenia symptom severity [102]; in Nepal, adapted for self- or caregiver report [94], adapted scoring cutoff > 10, sensitivity = 90%, positive items *α* = 0.82, negative items *α* = 0.88; combined *α* = 0.89. *PT*_*T0,T1,T2*_.
	*Alcohol Use Disorder Identification Test (AUDIT)*^B^: 13-item assessment of the quantity of alcohol consumption, tolerance, and dependence [103]; validated in a medical setting in Nepal [104]; cutoff ≥ 9, sensitivity 96.7% for males and 94.37% for females, specificity 79.6% and 85.4%, respectively; *α* = 0.82. *PT*_*T0,T1,T2*_.
Competency of provider	*Enhancing Assessment of Common Therapeutic factors (ENACT)-Patient version*^B^: 12-item patient version of the ENACT scale that allows patient and/or caregiver to comment on therapeutic rapport, comprehensiveness of mental health evaluation, and communication skills; developed in Nepal and shown to associate with depression treatment outcomes [105]. This is also an indicator of a positive experience of care [106]. *PT*_*T2*_.
Stigma and discrimination	*Discrimination and Stigma Scale Short Version (DISCUS)*^B^: 11-item scale to measure discrimination and stigma experienced by people with mental health problems [107]. *PT*_*T2*_.
	*Internalized Stigma of Mental Illness (ISMI)*^B^: 29-item scale used to measure internalized stigma among people with mental health problems [108]. *PT*_*T2*_.
Barriers to care	*Barriers to Access to Care Evaluation (BACE)*^B^: stigma and other barriers to accessing health services [109], adapted and used in rural Nepal *n* = 324 [47]. *PT*_*T2*_.
Cost of care	*Client Service Receipt Inventory (CSRI)*^B^: costs associated with psychiatric interventions [110]; records information on employment earnings and benefits, hospital care, primary care, social care, and support from informal caregivers (e.g., family); previously used in Nepal [111]. The period covered for costs will be from enrollment in the health facility onward until the assessment point (i.e., 0–3 months; 3–6 months). *PT*_*T1,T2*_.

Study objectives: ^A^primary outcome, ^B^secondary outcome, and ^C^mediator. Assessment time points. Primary care providers: *PCP*_*T0*_ = pre-training, *PCP*_*T1*_ = post-training, *PCP*_*T2*_ = 3-month follow-up, *PCP*_*T1*_ = 6-month follow-up. Patients: *PT*_*T0*_ = screening in primary care, *PT*_*T1*_ = 3-month follow-up, *PT*_*T2*_ = 6-month follow-up

Secondary outcomes at the PCP level for objective 1 include the Reported and Intended Behavior Scale (RIBS), which is a measure of behavioral intentions [84]; PCP knowledge about mental health conditions and their treatment, as assessed with selected questions from the mhGAP knowledge test [85–87]; clinical skill as assessed with a structured role play that is rated with the Enhancing Assessment of Common Therapeutic factors (ENACT) [88, 89], an observational tool with 15 competencies linked to “common factors” for good quality psychosocial care; and a supplementary competency assessment for 5 mhGAP competencies. In addition, after each structured role play, PCPs are asked about what diagnosis and treatment they would recommend for the standardized patient. In addition to explicit stigma, we will measure implicit stigma with the Implicit Association Test (IAT) adapted for Nepal [11]. A self-report of clinical self-efficacy is also collected [85–87].

The patient-level data will be evaluated in objective 2, which includes the implementation-effectiveness component. The primary implementation outcome is *reach*, operationalized as the accuracy of diagnosis. This will be established by recruiting patients from primary care facilities during months 4–6 post-training. The patients visiting the health facility will be evaluated by PCPs. After PCP evaluation, a researcher stationed in each health facility will screen the consenting patients with locally validated tools for PHQ-9 [67] for depression, the Generalized Anxiety Disorder (GAD-7) for anxiety, a version of the Positive and Negative Syndrome Scale (PANSS) [94] previously adapted for self or caregiver report in Nepal (the Nepali version includes screening items before proceeding to the 14 items), and the Alcohol Use Disorders Identification Test (AUDIT) [104].

If the person is identified by the PCP as having a mental health condition, the patient will be recruited for follow-up at the 3-month period. Similarly, a subset of patients visiting health facilities who are *not* detected as having mental health conditions by PCPs will be randomly selected and asked to participate as well. This will include a selection of both patients who score above and below screening cutoffs, with over recruitment of those screening above cutoffs, as they are more likely to have a missed diagnosis.

At 3 months after patient enrollment, a psychiatrist will complete a remote structured clinical interview using the Structured Clinical Interview for DSM-5-Research Version (SCID-5-RV) [91], including the modules for depression, generalized anxiety, mania, schizophrenia, and alcohol use disorder. The 3-month period between PCP diagnosis and psychiatrist SCID evaluation is because PCPs may do an initial assessment and then ask a patient to return in a few weeks to confirm the diagnosis. Therefore, PCPs have a 3-month window to confirm or revise their clinical diagnoses. The last recorded clinical diagnosis of the 3-month period will be the one compared against the psychiatrist’s SCID outcome.

**Fig. 4:**
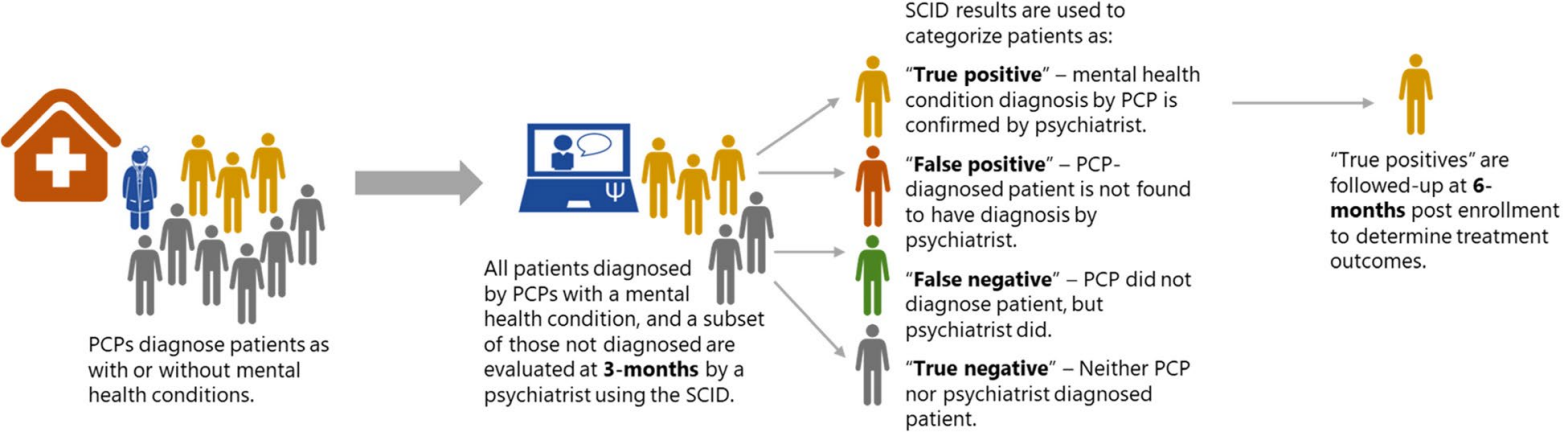
Data collection pathway for primary care providers (PCPs) and patients (PT) in primary care facilities. Abbreviations: mhGAP, mental health Gap Action Programme; RESHAPE, Reducing Stigma Among Healthcare Providers; IAU, implementation-as-usual. *Note*: Because our study can only recruit a subsample of those who do not receive a PCP diagnosis (estimated to be 40% in both arms) and 3-month follow-up can only include sub-samples of recruited patients who did not receive a diagnosis from a PCP (expected to be 10% of those who are true negatives and 50% of those who are false negatives), the between-arm comparison applies to a population which, compared to the general health facility-visiting population, has an overrepresentation of those who screen positive (yellow color participants at 3 months in the figure)

In the SCID, each mental health condition includes screening questions, and if screening questions are positive, the psychiatrist proceeds to a full battery for that module to make diagnoses according to the DSM-5 criteria. Of note, co-morbidities can be identified if participants meet the criteria for multiple disorders. From a feasibility perspective, it is not possible for psychiatrists to conduct a SCID on every patient seen by PCPs in a 3-month period. Therefore, the SCID interviews will be performed with all patients who received a mental health diagnosis by a PCP, as well as a subset of patients who did not receive a diagnosis, as mentioned above (see Fig. 4).

The psychiatrist is masked to the PCP diagnosis at the time of the SCID interview. After the SCID interview, the psychiatrist’s diagnosis will be used to categorize each patient into one of four groups: “true positives” = those patients who have the same diagnosis by the PCP and on the SCID; “false positives” = those patients who have a PCP diagnosis that is not confirmed on the SCID; “false negatives” = those patients who did not receive a PCP diagnosis, but who did receive a diagnosis on the SCID; and “true negatives” = those patients who were not diagnosed by a PCP and who did meet the criteria for any diagnosis on the SCID. We anticipate screening approximately 5500 patients in the primary care facilities and having approximately 1100 evaluated in SCID interviews by the psychiatrist. See Additional file 1: Fig. S3 for the estimated breakdown for recruitment based on categorization for the estimated breakdown for recruitment based on categorization according to PCP diagnoses, SCID interview outcomes and assumed sampling fractions. See Additional file 1 for details on *secondary outcomes*.

The participant timeline for PCPs and patients provides full details on when all instruments are administered (see Additional file 1: Table S1). Instruments have been piloted with PCPs and patients, and the time needed for completion at different time points was deemed feasible and acceptable to participants in the pilot cRCT.

### Recruitment

For objective 1, the participants are PCPs, and they will be recruited from the approximately 76 study health facilities in 24 municipalities. For recruitment, training, and supervision of PCPs, we will work together with municipalities and their health coordinators. Through our qualitative findings from previous studies, the travel allowances mandated by the Nepal government for PCPs to attend trainings and supervision act as an incentive and motivational factor for their retention and continued service delivery in health programs. Hence, we plan to comply with the government mandate by providing these travel allowances.

For objective 2, patients are recruited by research assistants at the primary care facility. For compensation, the patients will be provided with a household item such as soap, fruit, or a bag of sugar as a token of appreciation. This was deemed acceptable by PWLE and caregivers in the pilot cRCT.

### Assignment of interventions: allocation and sequence generation

The planned cRCT will randomize 24 municipalities to either IAU or RESHAPE using covariate-constrained randomization to achieve baseline covariate balance [117–119]. The cRCT will involve *multiple levels of clustering*: municipalities, health facilities within municipalities, PCPs within health facilities, and, for the patient-related outcomes of objective 2, patients within PCPs. At the time of randomization, the 24 municipalities will be randomized to either IAU or RESHAPE. Then, within each municipality, all enrolled health facilities will receive the implementation specific to that arm (i.e., IAU vs. RESHAPE). Please see Additional file 1 for details on the *concealment mechanism and implementation and assignment of interventions: masking and procedure for unmasking*.

### Plans for assessment, collection of outcomes, and promotion of retention and follow-up

PCP outcomes will be assessed on the first day of training, the last day of training, 3 months post-training, and again at 6 months post-training. The multiple time points are to allow for comparisons of immediate and sustained changes. Qualitative interviews with a subset of PCPs will be conducted approximately 12 months after mhGAP trainings. Please see Additional file 1 for additional details on the *collection of outcomes* and details on the *criteria for discontinuing or modifying allocated interventions, adherence to interventions, concomitant care, and post-trial care*.

### Eligibility criteria

#### Cluster eligibility

We selected municipalities in consultation with the Nepal Epidemiology and Disease Control Division of the Ministry of Health and Population at the federal level, the Ministry of Health and Population and the Province Health Directorate at the provincial level, and municipalities at the local level. The municipalities selected are representative of most rural and peri-urban regions of Nepal. See Additional file 1 for details on the *eligibility criteria* for PCPs and patients, *consent/assent procedures, confidentiality*, and *oversight and monitoring: data and safety monitoring board*.

### Sample size

The cRCT was designed with a minimum of 80% power for each of the two co-primary PCP outcomes (objective 1: stigma; objective 2: reach), respectively, at an overall 5% significance level. Given that we will consider RESHAPE a meaningful implementation strategy under the condition that both co-primary outcomes are statistically significant, we assumed a 5% two-tailed significance level for each outcome and calculated power using a closed-form sample size formula [120] that assumes that the generalized estimating equations (GEE) approach will be used for the analysis (identity link for stigma and log link for reach). For the purposes of these power calculations, we assumed the following sample sizes at each of the four levels: 24 municipalities randomized to one of two implementation arms (RESHAPE vs. IAU), 3 health facilities per municipality (72 in total), and 3 PCPs per health facility (of the total of 216, we assume 80% will provide data for objective 1 analyses at the 6-month timepoint).

For objective 2, we estimate that 1 PCP will deliver mental health services per facility (a total of 72 PCPs), and each will recruit approximately 80 patients (total approximate sample 5760), of which approximately 16 are followed up at 3 months (total 1152, see Additional file 1: Fig. S3). We note that, in practice, some health facilities may have more than 1 PCP who delivers MH services. Sensitivity analyses show that power is still high in this case when the 80 patients are divided between 2 or more PCPs (see Additional file 1: Table S2 and Table S3). See Additional file 1 for additional details on the *sample size, including assumptions on correlation parameters*.

### Statistical methods for primary and secondary outcomes

The main analysis of our two co-primary outcomes will be based on the intention-to-treat principle whereby all PCPs (objective 1 stigma) and all participants (objective 2 accurate diagnosis) will be included in the analysis in the study arm to which they were randomized irrespective of whether they complied with the assigned allocation of their municipality to one of the two implementation strategies (IAU and RESHAPE). Each analysis will be performed within the GEE framework paired with the matrix-adjusted equations (MAEE) approach [121] with identity link for stigma and log-link for reach (i.e., accuracy). MAEE is an approach whereby a set of estimating equations is posited for the correlation parameters just as estimating equations are used for the outcome model to estimate the impact of the RESHAPE implementation strategy. Importantly, MAEE provides confidence intervals for pairwise correlation parameters, which is particularly useful for the planning of future studies. Corrections such as the Kauermann-Carroll adjustment to variance estimates will be used to avoid “small-sample” bias that may arise given that fewer than 40 clusters (i.e., municipalities) are randomized [120, 122].

The model for each outcome will include the implementation arm, the covariates included in the constrained randomization procedure, and a set of PCP covariates identified a priori as potential confounders (e.g., age, gender, and health worker qualification level). If additional important covariates are identified post hoc, additional sensitivity analyses will add those covariates to the model, particularly if they are, by chance, imbalanced between arms. Please see Additional file 1 for additional details on the *statistical methods* as well as details on the *methods in analysis to handle protocol non-adherence and any statistical methods to handle missing data*, *interim analyses*, *methods for additional analyses*, *access to data and statistical code*, *data management*, and *dissemination plans*.

## Discussion

There are multiple public health and scientific potential benefits of this study. With current detection rates of mental illness by mhGAP-trained PCPs at less than 10% globally, an implementation strategy capable of doubling or tripling accurate detection would dramatically increase the number of people with mental illness entering care globally. With stigma from primary care workers against mental illness manifest as avoidance, discrimination, and reluctance to provide treatment, a successful intervention to reduce stigma could transform the care-seeking experiences of patients and make mental healthcare in primary care a normative global practice. Moreover, it is a matter of principle to have more inclusion of PWLE in the process of improving care.

### Trial status

Recruitment and training of PCPs began in February 2022, with patient recruitment planned to begin in June 2022. The Nepal Health Research Council conducted a study audit prior to the initiation of PCP trainings on 11 February 2022.

## Supplementary Information

**Additional file 1: Fig. S1.** RESHAPE study objectives and associated hypotheses. **Fig. S2.** Implementation science outcomes categorized according to RE-AIM framework. **Table S1.** Participant timeline: schedule of enrollment, interventions, and assessments for RESHAPE and IAU arms. **Fig. S3.** Key proportions for Objective 2 implementation power calculation at sample level. **Table S2.** Sensitivity of power to number of health workers per health facility - sample size assumptions. **Table S3.** Sensitivity of power to proportion of patients identified as HW-positive. **Fig. S4.** Decision tree model for cost-effectiveness analysis.

## Data Availability

Data will be made available through the United States National Institute of Mental Health Data Archive (https://nda.nih.gov/).

## Abbreviations

AUDIT: Alcohol Use Disorder Identification Test
BACE: Barriers to Access to Care Evaluation
cRCT: Cluster randomized controlled trial
CSRI: Client Service Receipt Inventory
DISCUS: Discrimination and Stigma Scale Short Version
DSMB: Data and Safety Monitoring Board
ENACT: Enhancing Assessment of Common Therapeutic factors
GAD-7: Generalized anxiety disorder
HIC: High-income countries
IAT: Implicit Association Tool
IAU: Implementation-as-usual
ISMI: Internalized stigma of mental health
LMICs: Low- and middle-income countries
mhGAP: Mental health Gap Action Programme
NIMH: National Institutes of Mental Health
PANSS: Positive and Negative Syndrome Scale
PCP: Primary care provider
PHQ-9: Patient Health Questionnaire-9
QALYs: Quality of life years
RIBS: Reported and Intended Behavior Scale
RE-AIM: Reach, Effectiveness, Adoption, Implementation, Maintenance
RESHAPE: Reducing Stigma Among Healthcare Providers
SCID-5-RV: Structured Clinical Interview for DSM-5-Research Version
TPO: Transcultural Psychosocial Organization
WHO: World Health Organization
WHODAS: WHO Disability Assessment Schedule

## References

[1] Thornicroft G, Chatterji S, Evans-Lacko S, Gruber M, Sampson N, Aguilar-Gaxiola S, Al-Hamzawi A, Alonso J, Andrade L, Borges G (2017). Undertreatment of people with major depressive disorder in 21 countries. Br J Psychiatry.

[2] Degenhardt L, Glantz M, Evans-Lacko S, Sadikova E, Sampson N, Thornicroft G, Aguilar-Gaxiola S, Al-Hamzawi A, Alonso J, Helena Andrade L (2017). Estimating treatment coverage for people with substance use disorders: an analysis of data from the World Mental Health Surveys. World Psychiatry.

[3] Alonso J, Liu Z, Evans-Lacko S, Sadikova E, Sampson N, Chatterji S, et al. Treatment gap for anxiety disorders is global: results of the World Mental Health Surveys in 21 countries. Depress Anxiety. 2018;35(3):195–208. 10.1002/da.22711 PMC6008788 29356216

[4] Fekadu A, Medhin G, Lund C, DeSilva M, Selamu M, Alem A, Asher L, Birhane R, Patel V, Hailemariam M (2019). The psychosis treatment gap and its consequences in rural Ethiopia. BMC Psychiatry.

[5] WHO (2010). mhGAP Intervention Guide for mental, neurological and substance-use disorders in non-specialized health settings: mental health Gap Action Programme (mhGAP).

[6] Fekadu A, Demissie M, Birhane R, Medhin G, Bitew T, Hailemariam M, Minaye A, Habtamu K, Milkias B, Petersen I (2022). Under detection of depression in primary care settings in low and middle-income countries: a systematic review and meta-analysis. Syst Rev.

[7] Jenkins R, Othieno C, Okeyo S, Kaseje D, Aruwa J, Oyugi H, Bassett P, Kauye F (2013). Short structured general mental health in service training programme in Kenya improves patient health and social outcomes but not detection of mental health problems-a pragmatic cluster randomised controlled trial. Int J Mental Health Syst.

[8] Fekadu A, Medhin G, Selamu M, Giorgis TW, Lund C, Alem A, Prince M, Hanlon C (2017). Recognition of depression by primary care clinicians in rural Ethiopia. BMC Fam Pract.

[9] Kauye F, Jenkins R, Rahman A (2014). Training primary health care workers in mental health and its impact on diagnoses of common mental disorders in primary care of a developing country, Malawi: a cluster-randomized controlled trial. Psychol Med.

[10] Jordans MJD, Luitel NP, Kohrt BA, Rathod SD, Garman EC, De Silva M, Komproe IH, Patel V, Lund C (2019). Community-, facility-, and individual-level outcomes of a district mental healthcare plan in a low-resource setting in Nepal: a population-based evaluation. PLoS Med.

[11] Kohrt BA, Jordans MJD, Turner EL, Rai S, Gurung D, Dhakal M, Bhardwaj A, Lamichhane J, Singla DR, Lund C (2021). Collaboration with people with lived experience of mental illness to reduce stigma and improve primary care services in Nepal: a pilot cluster randomized clinical trial. JAMA Netw Open.

[12] Luitel NP, Breuer E, Adhikari A, Kohrt BA, Lund C, Komproe IH, Jordans MJD (2020). Process evaluation of a district mental healthcare plan in Nepal: a mixed-methods case study. BJPsych Open.

[13] Keynejad R, Spagnolo J, Thornicroft G (2021). WHO mental health Gap Action Programme (mhGAP) intervention guide: updated systematic review on evidence and impact. Evidence-Based Mental Health..

[14] Heim E, Kohrt BA, Koschorke M, Milenova M, Thornicroft G. Reducing mental health related stigma in primary health care settings in low- and middle- income countries: a systematic review. Epidemiol Psychiatr Sci. 2018;29:e3. 10.1017/s2045796018000458. 10.1017/S2045796018000458 PMC6399081 30176952

[15] Vistorte AOR, Ribeiro WS, Jaen D, Jorge MR, Evans-Lacko S, Mari JJ (2018). Stigmatizing attitudes of primary care professionals towards people with mental disorders: a systematic review. Int J Psychiatry Med.

[16] Henderson C, Noblett J, Parke H, Clement S, Caffrey A, Gale-Grant O, Schulze B, Druss B, Thornicroft G (2014). Mental health-related stigma in health care and mental health-care settings. Lancet Psychiatry.

[17] Kakuma R, Minas H, van Ginneken N, Dal Poz MR, Desiraju K, Morris JE, Saxena S, Scheffler RM (2011). Human resources for mental health care: current situation and strategies for action. Lancet.

[18] Semrau M, Evans-Lacko S, Koschorke M, Ashenafi L, Thornicroft G (2015). Stigma and discrimination related to mental illness in low- and middle-income countries. Epidemiol Psychiatr Sci.

[19] Jenkins R, Othieno C, Okeyo S, Aruwa J, Kingora J, Jenkins B (2013). Health system challenges to integration of mental health delivery in primary care in Kenya - perspectives of primary care health workers. BMC Health Serv Res.

[20] Muga FA, Jenkins R (2008). Training, attitudes and practice of district health workers in Kenya. Soc Psychiatry Psychiatr Epidemiol.

[21] Koschorke M, Oexle N, Ouali U, Cherian AV, Deepika V, Mendon GB, Gurung D, Kondratova L, Muller M, Lanfredi M (2021). Perspectives of healthcare providers, service users, and family members about mental illness stigma in primary care settings: a multi-site qualitative study of seven countries in Africa, Asia, and Europe. PLoS One.

[22] Griffith JL, Kohrt BA (2016). Managing stigma effectively: what social psychology and social neuroscience can teach us. Acad Psychiatry.

[23] Ola B, Crabb J, Adewuya A, Olugbile F, Abosede OA. The state of readiness of Lagos State Primary Health Care Physicians to embrace the care of depression in Nigeria. Community Ment Health J. 2014;50(2):239–44. 10.1007/s10597-013-9648-9 23912148

[24] Gwaikolo WS, Kohrt BA, Cooper JL (2017). Health system preparedness for integration of mental health services in rural Liberia. BMC Health Serv Res.

[25] Kohrt BA, Harper I (2008). Navigating diagnoses: understanding mind-body relations, mental health, and stigma in Nepal. Cult Med Psychiatry.

[26] Nyblade L, Stangl A, Weiss E, Ashburn K (2009). Combating HIV stigma in health care settings: what works?. J Int AIDS Soc.

[27] Nyblade L (2016). Disentangling stigma to find entry points for intervention. Sex Health Exch.

[28] Khuat THO, Ashburn K, Pulerwitz J, Ogden J, Nyblade L (2008). Improving hospital-based quality of care in Vietnam by reducing HIV-related stigma and discrimination.

[29] Batey DS, Whitfield S, Mulla M, Stringer KL, Durojaiye M, McCormick L, Turan B, Nyblade L, Kempf MC, Turan JM (2016). Adaptation and implementation of an intervention to reduce HIV-related stigma among healthcare workers in the United States: piloting of the FRESH Workshop. AIDS Patient Care STDS.

[30] Srithanaviboonchai K, Stockton M, Pudpong N, Chariyalertsak S, Prakongsai P, Chariyalertsak C, Smutraprapoot P, Nyblade L (2017). Building the evidence base for stigma and discrimination-reduction programming in Thailand: development of tools to measure healthcare stigma and discrimination. BMC Public Health.

[31] Kohrt BA, Jordans MJD, Turner EL, Sikkema KJ, Luitel NP, Rai S, Singla DR, Lamichhane J, Lund C, Patel V (2018). Reducing stigma among healthcare providers to improve mental health services (RESHAPE): protocol for a pilot cluster randomized controlled trial of a stigma reduction intervention for training primary healthcare workers in Nepal. Pilot Feasibility Stud.

[32] Kohrt BA, Turner EL, Rai S, Bhardwaj A, Sikkema KJ, Adelekun A, Dhakal M, Luitel NP, Lund C, Patel V (2020). Reducing mental illness stigma in healthcare settings: proof of concept for a social contact intervention to address what matters most for primary care providers. Soc Sci Med.

[33] Kohrt BA, Ottman K, Panter-Brick C, Konner M, Patel V. Why we heal: the evolution of psychological healing and implications for global mental health. Clin Psychol Rev. 2020;82:101920. 10.1016/j.cpr.2020.101920. 10.1016/j.cpr.2020.101920 33126037

[34] Kohrt BA, Kirmayer LJ, Worthman CM, Kitayama S, Lemelson R, Cummings CA (2020). Social neuroscience in global mental health: case study on stigma reduction in Nepal. Culture, mind, and brain: emerging concepts, models, and applications.

[35] Wang C, Burris MA (1997). Photovoice: concept, methodology, and use for participatory needs assessment. Health Educ Behav.

[36] Russinova Z, Mizock L, Bloch P (2018). Photovoice as a tool to understand the experience of stigma among individuals with serious mental illnesses. Stigma Health.

[37] Knaak S, Modgill G, Patten SB (2014). Key ingredients of anti-stigma programs for health care providers: a data synthesis of evaluative studies. Can J Psychiatry Rev Can Psychiatrie.

[38] Bhardwaj A, Gurung D, Rai S, Kaiser BN, Tergesen C, Sikkema KJ, et al. Treatment preferences for pharmacological versus psychological interventions among primary care providers in Nepal: Mixed methods analysis of a pilot cluster randomized controlled trial. Int J Environ Res Public Health. 2022;19(4):2149. 10.3390/ijerph19042149. 10.3390/ijerph19042149 PMC8871897 35206331

[39] Rai S, Gurung D, Kaiser BN, Sikkema KJ, Dhakal M, Bhardwaj A, Tergesen C, Kohrt BA (2018). A service user co-facilitated intervention to reduce mental illness stigma among primary healthcare workers: utilizing perspectives of family members and caregivers. Fam Syst Health.

[40] Kaiser BN, Varma S, Carpenter-Song E, Sareff R, Rai S, Kohrt BA (2020). Eliciting recovery narratives in global mental health: benefits and potential harms in service user participation. Psychiatr Rehabil J.

[41] Bhardwaj A, Gurung D, Rai S, Kaiser BN, Cafaro CL, Sikkema KJ, Lund C, Luitel NP, Kohrt BA (2022). Treatment preferences for pharmacological versus psychological interventions among primary care providers in Nepal: mixed methods analysis of a pilot cluster randomized controlled trial. Int J Environ Res Public Health.

[42] Bogardus ES (1925). Measuring social distance. J Appl Sociol.

[43] Gaglio B, Shoup JA, Glasgow RE (2013). The RE-AIM Framework: a systematic review of use over time. Am J Public Health.

[44] Glasgow RE, Vogt TM, Boles SM (1999). Evaluating the public health impact of health promotion interventions: the RE-AIM framework. Am J Public Health.

[45] Proctor E, Silmere H, Raghavan R, Hovmand P, Aarons G, Bunger A, Griffey R, Hensley M (2011). Outcomes for implementation research: conceptual distinctions, measurement challenges, and research agenda. Admin Policy Mental Health Mental Health Serv Res.

[46] Glasgow RE, Estabrooks PE (2018). Pragmatic applications of RE-AIM for health care initiatives in community and clinical settings. Prev Chronic Dis.

[47] Luitel NP, Jordans MJD, Kohrt BA, Rathod SD, Komproe IH (2017). Treatment gap and barriers for mental health care: a cross-sectional community survey in Nepal. PLoS One.

[48] Rai Y, Gurung D, Gautam K (2021). Insight and challenges: mental health services in Nepal. BJPsych Int.

[49] Kohrt BA, Jordans MJD, Tol WA, Speckman RA, Maharjan SM, Worthman CM, Komproe IH (2008). Comparison of mental health between former child soldiers and children never conscripted by armed groups in Nepal. Jama.

[50] Kohrt BA (2009). Vulnerable social groups in post-conflict settings: a mixed-methods policy analysis and epidemiology study of caste and psychological morbidity in Nepal. Interv: Int J Mental Health, Psychosoc Work Couns Areas Armed Conflict.

[51] Kohrt BA, Worthman CM (2009). Gender and anxiety in Nepal: the role of social support, stressful life events, and structural violence. CNS Neurosci Ther.

[52] Kohrt BA, Jordans MJ, Tol WA, Perera E, Karki R, Koirala S, Upadhaya N (2010). Social ecology of child soldiers: child, family, and community determinants of mental health, psychosocial well-being, and reintegration in Nepal. Transcult Psychiatry.

[53] Tol WA, Kohrt BA, Jordans MJD, Thapa SB, Pettigrew J, Upadhaya N, de Jong JTVM (2010). Political violence and mental health: a multi-disciplinary review of the literature on Nepal. Soc Sci Med.

[54] Kohrt BA, Hruschka DJ, Worthman CM, Kunz RD, Baldwin JL, Upadhaya N, Acharya NR, Koirala S, Thapa SB, Tol WA (2012). Political violence and mental health in Nepal: prospective study. Br J Psychiatry.

[55] Haviland MJ, Shrestha A, Decker MR, Kohrt BA, Kafle HM, Lohani S, Thapa L, Surkan PJ (2014). Barriers to sexual and reproductive health care among widows in Nepal. Int J Gynaecol Obstet.

[56] Upadhaya N, Luitel NP, Koirala S, Adhikari RP, Gurung D, Shrestha P, Tol WA, Kohrt BA, Jordans MJD (2014). The role of mental health and psychosocial support nongovernmental organizations: reflections from post-conflict Nepal. Interv: Int J Mental Health, Psychosoc Work Couns Areas Armed Conflict.

[57] Kohrt BA, Bourey C (2016). Culture and comorbidity: intimate partner violence as a common risk factor for maternal mental illness and reproductive health problems among former child soldiers in Nepal. Med Anthropol Q.

[58] Angdembe M, Kohrt BA, Jordans M, Rimal D, Luitel NP (2017). Situational analysis to inform development of primary care and community-based mental health services for severe mental disorders in Nepal. Int J Mental Health Syst.

[59] Hagaman AK, Khadka S, Lohani S, Kohrt B (2017). Suicide in Nepal: a modified psychological autopsy investigation from randomly selected police cases between 2013 and 2015. Soc Psychiatry Psychiatr Epidemiol.

[60] Jordans M, Rathod S, Fekadu A, Medhin G, Kigozi F, Kohrt B, et al. Suicidal ideation and behaviour among community and health care seeking populations in five low- and middle-income countries: a cross-sectional study. Epidemiol Psychiatr Sci. 2018;27(4):393–402. 10.1017/S2045796017000038 PMC5559346 28202089

[61] Kane JC, Luitel NP, Jordans MJD, Kohrt BA, Weissbecker I, Tol WA. Mental health and psychosocial problems in the aftermath of the Nepal earthquakes: findings from a representative cluster sample survey. Epidemiol Psychiatr Sci. 2018;27(3):301–10. 10.1017/S2045796016001104 PMC5502203 28065208

[62] Bhardwaj A, Bourey C, Rai S, Adhikari RP, Worthman CM, Kohrt BA (2018). Interpersonal violence and suicidality among former child soldiers and war-exposed civilian children in Nepal. Global Mental Health.

[63] Hagaman AK, Khadka S, Wutich A, Lohani S, Kohrt BA (2018). Suicide in Nepal: qualitative findings from a modified case-series psychological autopsy investigation of suicide deaths. Cult Med Psychiatry.

[64] Luitel NP, Baron EC, Kohrt BA, Komproe IH, Jordans MJD (2018). Prevalence and correlates of depression and alcohol use disorder among adults attending primary health care services in Nepal: a cross sectional study. BMC Health Serv Res.

[65] Kohrt BA, Carruth L. Syndemic effects in complex humanitarian emergencies: a framework for understanding political violence and improving multi-morbidity health outcomes. Soc Sci Med. 2022;295:113378. 10.1016/j.socscimed.2020.113378. 10.1016/j.socscimed.2020.113378 PMC7501533 33051023

[66] Peoples N, Gong E, Gautam K, Khanal SN, Kohrt BA, Koirala S, Amatya A, Xiong S, Østbye T, Moe J (2021). Perception and use of primary healthcare services among people with cardiometabolic diseases in two resource-limited areas in Nepal: a mixed methods study. Front Public Health.

[67] Kohrt BA, Luitel NP, Acharya P, Jordans MJD (2016). Detection of depression in low resource settings: validation of the Patient Health Questionnaire (PHQ-9) and cultural concepts of distress in Nepal. BMC Psychiatry.

[68] Neupane D, Panthi B, McLachlan CS, Mishra SR, Kohrt BA, Kallestrup P (2015). Prevalence of undiagnosed depression among persons with hypertension and associated risk factors: a cross-sectional study in urban Nepal. PLoS One.

[69] Niraula K, Kohrt B, Flora M, Thapa N, Mumu S, Pathak R, Stray-Pedersen B, Ghimire P, Regmi B, MacFarlane E (2013). Prevalence of depression and associated risk factors among persons with type-2 diabetes mellitus without a prior psychiatric history: a cross-sectional study in clinical settings in urban Nepal. BMC Psychiatry.

[70] Luitel NP, Jordans MJ, Sapkota RP, Tol WA, Kohrt BA, Thapa SB, Komproe IH, Sharma B (2013). Conflict and mental health: a cross-sectional epidemiological study in Nepal. Soc Psychiatry Psychiatr Epidemiol.

[71] Kohrt BA, Kunz RD, Baldwin JL, Koirala NR, Sharma VD, Nepal MK (2005). “Somatization” and “comorbidity”: a study of Jhum-Jhum and depression in rural Nepal. Ethos.

[72] Suvedi BK, Pradhan A, Barnett S, Puri M, Chitrakar SR, Poudel P, Sharma S, Hulton L (2009). Nepal maternal mortality and morbidity study 2008/2009: summary of preliminary findings.

[73] Ministry of Health and Population (2021). National mental health strategy & action plan 2077.

[74] World Health Organization (2016). mhGAP intervention guide for mental, neurological and substance use disorders in non-specialized health settings: mental health Gap Action Programme (mhGAP) – version 2.0.

[75] World Health Organization (2017). mhGAP training manuals: for the mhGAP intervention guide for mental, neurological and substance use disorders in non-specialized health settings – version 2.0 (for field testing).

[76] Jordans MJ, Luitel NP, Pokhrel P, Patel V (2016). Development and pilot testing of a mental healthcare plan in Nepal. Br J Psychiatry.

[77] Tergesen CL, Gurung D, Dhungana S, Risal A, Basel P, Tamrakar D, Amatya A, Park LP, Kohrt BA (2021). Impact of service user video presentations on explicit and implicit stigma toward mental illness among medical students in Nepal: a randomized controlled trial. Int J Environ Res Public Health.

[78] Link BG, Yang LH, Phelan JC, Collins PY (2004). Measuring mental illness stigma. Schizophr Bull.

[79] Pescosolido BA, Medina TR, Martin JK, Long JS (2013). The “backbone” of stigma: identifying the global core of public prejudice associated with mental illness. Am J Public Health.

[80] Lincoln TM, Arens E, Berger C, Rief W (2008). Can antistigma campaigns be improved? A test of the impact of biogenetic vs psychosocial causal explanations on implicit and explicit attitudes to schizophrenia. Schizophr Bull.

[81] Rüsch N, Corrigan PW, Todd AR, Bodenhausen GV (2010). Implicit self-stigma in people with mental illness. J Nerv Ment Dis.

[82] Cooper LA, Roter DL, Carson KA, Beach MC, Sabin JA, Greenwald AG, Inui TS (2012). The associations of clinicians’ implicit attitudes about race with medical visit communication and patient ratings of interpersonal care. Am J Public Health.

[83] Wang X, Huang X, Jackson T, Chen R (2012). Components of implicit stigma against mental illness among Chinese students. PLoS One.

[84] Evans-Lacko S, Rose D, Little K, Flach C, Rhydderch D, Henderson C, Thornicroft G (2011). Development and psychometric properties of the Reported and Intended Behaviour Scale (RIBS): a stigma-related behaviour measure. Epidemiol Psychiatr Sci.

[85] Mutiso VN, Pike KM, Musyimi CN, Rebello TJ, Tele A, Gitonga I, et al. Changing patterns of mental health knowledge in rural Kenya after intervention using the WHO mhGAP-Intervention Guide. Psychol Med. 2018;12(1):57. 10.1017/S0033291718003112 30345938

[86] Spagnolo J, Champagne F, Leduc N, Rivard M, Piat M, Laporta M, Melki W, Charfi F (2018). Mental health knowledge, attitudes, and self-efficacy among primary care physicians working in the Greater Tunis area of Tunisia. Int J Mental Health Syst.

[87] Keynejad RC, Dua T, Barbui C, Thornicroft G (2018). WHO Mental Health Gap Action Programme (mhGAP) Intervention Guide: a systematic review of evidence from low and middle-income countries. Evid Based Ment Health.

[88] Kohrt BA, Jordans MJD, Rai S, Shrestha P, Luitel NP, Ramaiya MK, Singla DR, Patel V (2015). Therapist competence in global mental health: development of the Enhancing Assessment of Common Therapeutic factors (ENACT) rating scale. Behav Res Ther.

[89] Kohrt BA, Ramaiya MK, Rai S, Bhardwaj A, Jordans MJD (2015). Development of a scoring system for non-specialist ratings of clinical competence in global mental health: a qualitative process evaluation of the Enhancing Assessment of Common Therapeutic factors (ENACT) scale. Global Mental Health.

[90] Kohrt BA, Schafer A, Willhoite A, van’t Hof E, Pedersen GA, Watts S, Ottman K, Carswell K, van Ommeren M (2020). Ensuring Quality in Psychological Support (WHO EQUIP): developing a competent global workforce. World Psychiatry.

[91] First MB, Williams JBW, Karg RS, Spitzer RL. SCID-5-CV: structured clinical interview for DSM-5 disorders, clinician version. Washington, DC: American Psychiatric Association Publishing; 2015.

[92] Ghimire DJ, Chardoul S, Kessler RC, Axinn WG, Adhikari BP (2013). Modifying and validating the Composite International Diagnostic Interview (CIDI) for use in Nepal. Int J Methods Psychiatr Res.

[93] World Health Organization (2000). World Health Organization disability assessment schedule: WHODAS II. Phase 2 field trials. Health services research.

[94] Jordans MJD, Aldridge L, Luitel NP, Baingana F, Kohrt BA (2017). Evaluation of outcomes for psychosis and epilepsy treatment delivered by primary health care workers in Nepal: a cohort study. Int J Mental Health Syst.

[95] Tol WA, Komproe IH, Jordans MJD, Thapa SB, Sharma B, De Jong JTVM (2009). Brief multi-disciplinary treatment for torture survivors in Nepal: a naturalistic comparative study. Int J Soc Psychiatry.

[96] Sangraula M, van’t Hof E, Luitel NP, Turner EL, Marahatta K, Nakao JH, van Ommeren M, MJD J, Kohrt BA (2018). Protocol for a feasibility study of group-based focused psychosocial support to improve the psychosocial well-being and functioning of adults affected by humanitarian crises in Nepal: Group Problem Management Plus (PM+). Pilot Feasibility Stud.

[97] Pattanaphesaj J, Thavorncharoensap M, Ramos-Goñi JM, Tongsiri S, Ingsrisawang L, Teerawattananon Y (2018). The EQ-5D-5L valuation study in Thailand. Expert Rev Pharmacoecon Outcomes Res.

[98] Kroenke K, Spitzer RL, Williams JBW (2001). The PHQ-9. J Gen Intern Med.

[99] Dangal MR, Bajracharya LS (2020). Students anxiety experiences during COVID-19 in Nepal. Kathmandu Univ Med J.

[100] Adhikari SP, Rawal N, Shrestha DB, Budhathoki P, Banmala S, Awal S, Bhandari G, Poudel R, Parajuli AR (2021). Prevalence of anxiety, depression, and perceived stigma in healthcare workers in Nepal during later phase of first wave of COVID-19 pandemic: a web-based cross-sectional survey. Cureus.

[101] Sharma K, Dhungana G, Adhikari S, Bista Pandey A, Sharma M (2021). Depression and anxiety among patients with type II diabetes mellitus in Chitwan Medical College Teaching Hospital, Nepal. Nurs Res Pract.

[102] Kay SR, Fiszbein A, Opler LA (1987). The Positive and Negative Syndrome Scale (PANSS) for schizophrenia. Schizophr Bull.

[103] Saunders JB, Aasland OG, Babor TF, de la Fuente JR, Grant M (1993). Development of the Alcohol Use Disorders Identification Test (AUDIT): WHO Collaborative Project on Early Detection of Persons with Harmful Alcohol Consumption--II. Addiction.

[104] Pradhan B, Chappuis F, Baral D, Karki P, Rijal S, Hadengue A, Gache P (2012). The Alcohol Use Disorders Identification Test (AUDIT): validation of a Nepali version for the detection of alcohol use disorders and hazardous drinking in medical settings. Subst Abuse Treat Prev Policy.

[105] Jordans MJD, Luitel NP, Garman E, Kohrt BA, Rathod SD, Shrestha P, Komproe IH, Lund C, Patel V (2019). Effectiveness of psychological treatments for depression and alcohol use disorder delivered by community-based counsellors: two pragmatic randomised controlled trials within primary healthcare in Nepal. Br J Psychiatry.

[106] Larson E, Leslie HH, Kruk ME (2017). The determinants and outcomes of good provider communication: a cross-sectional study in seven African countries. BMJ Open.

[107] Brohan E, Clement S, Rose D, Sartorius N, Slade M, Thornicroft G (2013). Development and psychometric evaluation of the Discrimination and Stigma Scale (DISC). Psychiatry Res.

[108] Ritsher JB, Otilingam PG, Grajales M (2003). Internalized stigma of mental illness: psychometric properties of a new measure. Psychiatry Res.

[109] Clement S, Brohan E, Jeffery D, Henderson C, Hatch SL, Thornicroft G (2012). Development and psychometric properties the Barriers to Access to Care Evaluation scale (BACE) related to people with mental ill health. BMC Psychiatry.

[110] Beecham J, Knapp M (2001). Costing psychiatric interventions. Meas Mental Health Needs.

[111] De Silva MJ, Rathod SD, Hanlon C, Breuer E, Chisholm D, Fekadu A, Jordans M, Kigozi F, Petersen I, Shidhaye R (2016). Evaluation of district mental healthcare plans: the PRIME consortium methodology. Br J Psychiatry.

[112] Parrillo VN, Donoghue C (2005). Updating the Bogardus social distance studies: a new national survey. Soc Sci J.

[113] Link BG. Understanding labeling effects in the area of mental disorders: an assessment of the effects of expectations of rejection. Am Sociol Rev. 1987;52(1):96–112.

[114] Peters RM, Van Brakel WH, Zweekhorst MB, Damayanti R, Bunders JF (2014). The cultural validation of two scales to assess social stigma in leprosy. PLoS Negl Trop Dis.

[115] van Brakel WH, Cataldo J, Grover S, Kohrt BA, Nyblade L, Stockton M, Wouters E, Yang LH (2019). Out of the silos: identifying cross-cutting features of health-related stigma to advance measurement and intervention. BMC Med.

[116] Kohrt BA, Mutamba BB, Luitel NP, Gwaikolo W, Onyango Mangen P, Nakku J, Rose K, Cooper J, Jordans MJD, Baingana F (2018). How competent are non-specialists trained to integrate mental health services in primary care? Global health perspectives from Uganda, Liberia, and Nepal. Int Rev Psychiatry.

[117] Li F, Lokhnygina Y, Murray DM, Heagerty PJ, DeLong ER (2016). An evaluation of constrained randomization for the design and analysis of group-randomized trials. Stat Med.

[118] Gallis JA, Li F, Yu H, Turner EL (2018). cvcrand and cptest: commands for efficient design and analysis of cluster randomized trials using constrained randomization and permutation tests. Stata J.

[119] Turner EL, Prague M, Gallis JA, Li F, Murray DM (2017). Review of recent methodological developments in group-randomized trials: part 2-analysis. Am J Public Health.

[120] Wang X, Turner EL, Preisser JS, Li F. Power considerations for generalized estimating equations analyses of four-level cluster randomized trials. Biom J. 2022;64(4):663–80. 10.1002/bimj.202100081 PMC9574475 34897793

[121] Preisser JS, Lu B, Qaqish BF (2008). Finite sample adjustments in estimating equations and covariance estimators for intracluster correlations. Stat Med.

[122] Kauermann G, Carroll RJ (2001). A note on the efficiency of sandwich covariance matrix estimation. J Am Stat Assoc.

