# Supplementary material for: Implementation strategy in collaboration with people with lived experience of mental illness to reduce stigma among primary care providers in Nepal (RESHAPE): protocol for a type 3 hybrid implementation effectiveness cluster randomized controlled trial
Source: Implement Sci. 2022 Jun 16;17:39. doi: 10.1186/s13012-022-01202-x (PMC9205129; doi:10.1186/s13012-022-01202-x)
Supplement: Supplementary file 1 — Additional file 1: Fig. S1. RESHAPE study objectives and associated hypotheses. Fig. S2. Implementation science outcomes categorized according to RE-AIM framework. Table S1. Participant timeline: schedule of enrollment, interventions, and assessments for RESHAPE and IAU arms. Fig. S3. Key proportions for Objective 2 implementation power calculation at sample level. Table S2. Sensitivity of power to number of health workers per health facility - sample size assumptions. Table S3. Sensitivity of power to proportion of patients identified as HW-positive. Fig. S4. Decision tree model for cost-effectiveness analysis. [file 13012_2022_1202_MOESM1_ESM.docx]

Additional file 1


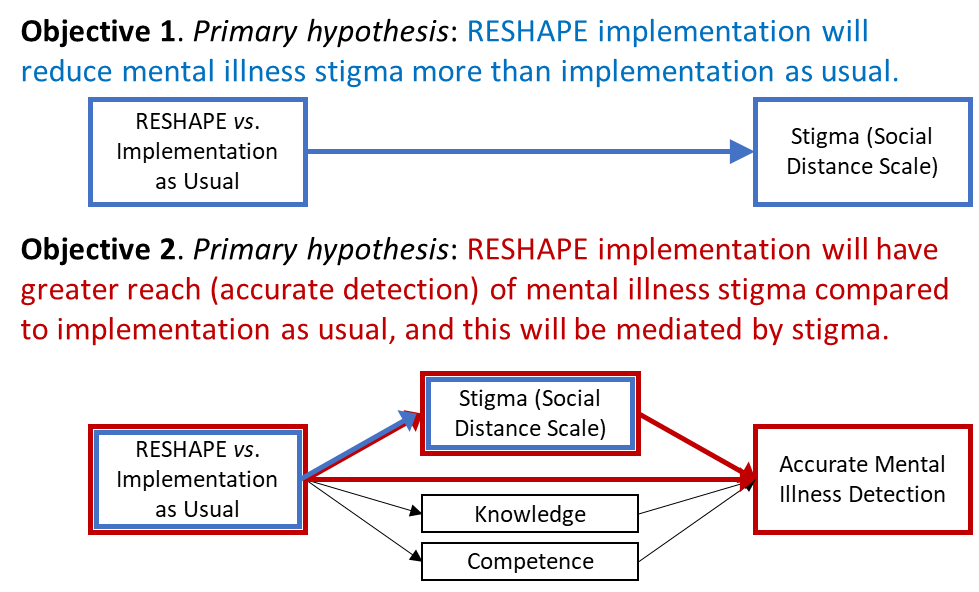


**Figure S1**. RESHAPE study objectives and associated hypotheses.


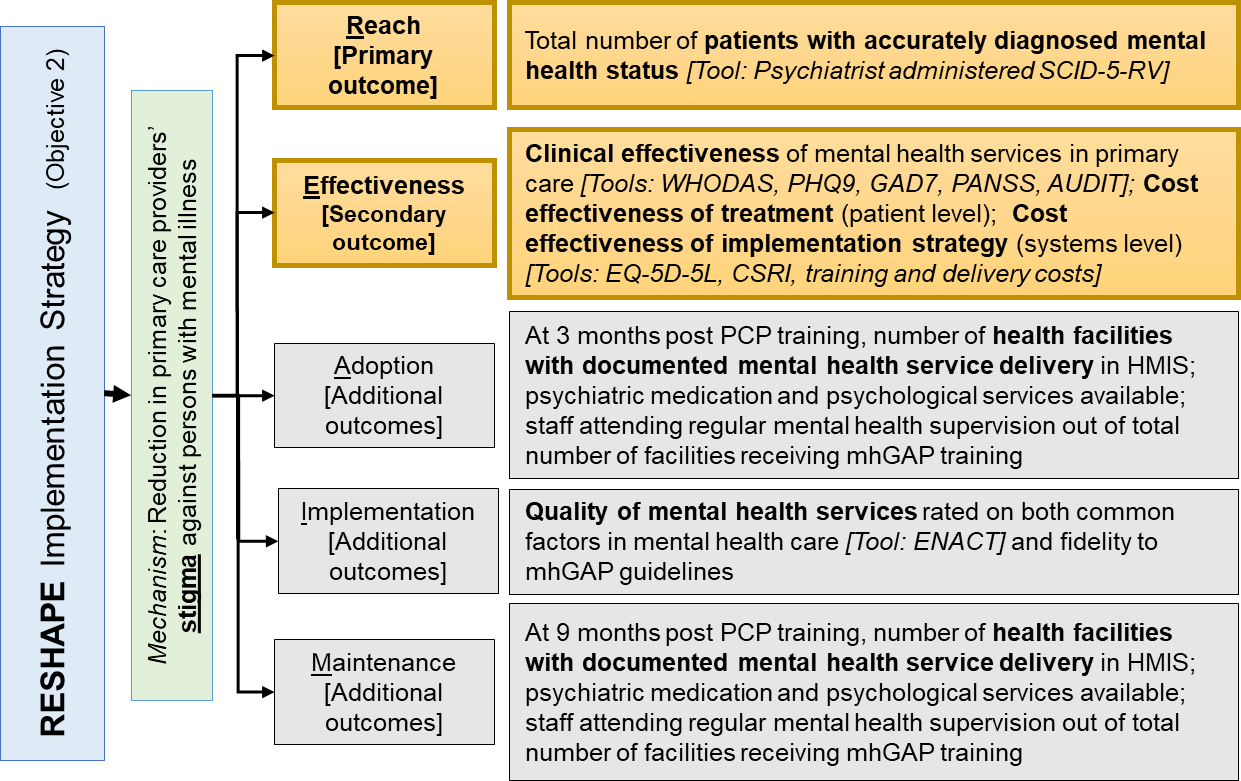


**Figure S2**. Implementation science outcomes categorized according to RE-AIM framework. Abbreviations: SCID-5-RV, Structured Clinical Interview for DSM5—Research Version; WHODAS, World Health Organization Disability Assessment Schedule; PHQ9, Patient Health Questionnaire; GAD7, Generalized Anxiety Disorder; PANSS, Positive and Negative Syndrome Scale; EQ-5D-5L, EuroQol 5 Dimension quality of life scale; HMIS, Health Management Information System; ENACT, Enhancing Assessment of Common Therapeutic Factors.

**Study setting and healthcare workforce in Nepal** (additional details)

In the government health system, the health workforce is managed at the provincial and municipal levels. At the central level, curricula for health workers, including mental health curricula, are approved by the National Health Training Center (NHTC). Primary health centers and health posts are established in each electoral area as a first referral point. Health posts and community health units are the lowest level facilities functioning in the community. There are approximately 3-9 primary care facilities (including primary health centers, health posts, etc.) in each municipality. The primary care health workforce can be divided between ‘prescribers’ (i.e., medical officers, health assistants and auxiliary health workers) who have prescription rights as per the Government of Nepal and ‘non-prescribers’ (i.e., staff nurses and auxiliary nurse midwives) who do not have the prescription rights. This study will focus on those PCPs who have prescribing rights (medical officers, health assistants, and auxiliary health workers). The prescribing PCPs in Nepal have been delivering services through primary health centers and health posts. Health assistants and auxiliary health workers complete 3-year and 18-month courses after high-school equivalent respectively. Medical officers are MBBS-level doctors.

**Outcomes** (additional details)

A secondary outcome for Objective 2 is clinical effectiveness of treatment provided by PCPs. This will be measured with symptom specific tools with interviewer-administered tools, which are self-report tools (not clinician-administered diagnostic tools): PHQ-9 [1] for depression, GAD-7 for anxiety, PANSS [2] for psychosis, and AUDIT [3] for alcohol use disorder. All tools are administered to the selected patients presenting at primary care facilities. Because all tools are used, potential co-morbidities will be identified. Functioning will be assessed with the World Health Organization Disability Assessment Schedule (WHODAS-II) [4]. Quality of life years (QALYs) will be assessed with the EQ-5D-5L [5]. Of note, for any tools completed with input by the caregiver, this information will be recorded. Other measures, which are collected only in the subset of patients followed up in the clinical diagnostic interview, will include costs of care for cost-effectiveness analysis, measured with an adapted version of the Client Service Receipt Inventory (CSRI) [6], barriers to care including stigma measured with the Barriers to Care Assessment (BACE) [7] and patient level stigma measured with the Discrimination and Stigma Scale Short Version (DISCUS) [8] and Internalized Stigma of Mental Illness (ISMI) [9]. These measures will also be research assistant administered tools collected at the 3- and 6-month follow-ups.

**Table S1. Participant timeline:** schedule of enrollment, interventions, and assessments for RESHAPE and IAU arms.

|  | **STUDY PERIOD** | | | | | | | | |  |
| --- | --- | --- | --- | --- | --- | --- | --- | --- | --- | --- |
| **PRIMARY CARE PROVIDERS** – trained to deliver mental health services in IAU or RESHAPE implementation strategy | | | | | | | | | | |
|  | **Cluster Allocation** | **Enrollment** | **Post-allocation** | | | | | | | **Close-out** |
| **TIMEPOINT** | ***-t_1_*** | ***t_0_*** | ***t_1_*** | ***t_2_*** | ***t_3_*** | ***t_4_*** | ***t_5_*** | ***t_6_*** | ***t_7_*** | ***t_8_*** |
| **Month** | *0* | *1* | *1* | *1* | *3* |  |  | *6* |  | *11* |
| **Primary Care Provider  Data Collection Point** |  | ***PCP_Screen_*** | ***PCP_T0_*** | ***PCP_T1_*** | ***PCP_T2_*** |  |  | ***PCP_T3_*** |  |  |
| **ENROLLMENT:** |  | | | | | | | |  |  |
| Allocation | X |  |  |  |  |  |  |  |  |  |
| Eligibility screen |  | X |  |  |  |  |  |  |  |  |
| Informed consent |  | X |  |  |  |  |  |  |  |  |
| **INTERVENTIONS:** |  | | | | | | | |  |  |
| mhGAP training |  |  | <---------🡪 | |  |  |  |  |  |  |
| Supervision and health facility management |  |  |  |  | <------------------------------------------🡪 | | | | |  |
| **ASSESSMENTS:** |  | | | | | | | |  |  |
| *Social Distance Scale (SDS)* |  |  | X | X | X |  |  | X |  |  |
| *Reported and Intended Behavior Scale (RIBS)* |  |  | X | X | X |  |  | X |  |  |
| *Implicit Association Test (IAT)* |  |  | X | X | X |  |  | X |  |  |
| *mhGAP Knowledge* |  |  | X | X | X |  |  | X |  |  |
| *Observed competency (ENACT)* |  |  | X | X | X |  |  | X |  |  |
| *mhGAP Self-Efficacy* |  |  | X | X | X |  |  | X |  |  |
| *Qualitative interviews* |  |  |  |  |  |  |  |  |  | X |
| **PATIENTS**– patients treated by IAU or RESHAPE-trained primary healthcare workers | | | | | | | | | | |
|  | **Cluster Allocation** |  | **Post-allocation** | | | | | | | **Close-out** |
| **TIMEPOINT** | ***-t_1_*** |  | ***t_1_*** | ***t_2_*** | ***t_3_*** | ***t_4_*** | ***t_5_*** | ***t_6_*** | ***t_7_*** | ***t_8_*** |
| **Month** | *0* |  |  |  |  | *3-5* | *3-5* |  | *6-8* | *9-11* |
| **Patient Data Collection Point** |  |  |  |  |  | ***PT Screen*** | ***PT_T0_*** |  | ***PT_T1_*** | ***PT_T2_*** |
| **ENROLLMENT:** |  | | | | | | | | | |
| Allocation | X |  |  |  |  |  |  |  |  |  |
| Eligibility screen |  |  |  |  |  | X |  |  |  |  |
| Informed consent |  |  |  |  |  | X |  |  |  |  |
| **INTERVENTIONS:** |  | | | | | | | |  |  |
| mhGAP-based diagnosis |  |  |  |  |  | <-> |  |  |  |  |
| mhGAP-based medication management |  |  |  |  |  |  | <----------------------🡪 | | |  |
| Psychological treatments (HAP/CAP) |  |  |  |  |  |  | <----------------------🡪 | | |  |
| **ASSESSMENTS:** |  | | | | | | | | | |
| Research Assistant Administered |  | | | | | | | | | |
| *Patient – WHODAS* |  |  |  |  |  |  | X |  | X | X |
| *Patient – PHQ9* |  |  |  |  |  |  | X |  | X | X |
| *Patient – GAD7* |  |  |  |  |  |  | X |  | X | X |
| *Patient – PANSS* |  |  |  |  |  |  | X |  | X | X |
| *Patient – AUDIT* |  |  |  |  |  |  | X |  | X | X |
| *Patient – EQ-5D-5L* |  |  |  |  |  |  | X |  | X | X |
| *Patient – ENACT* |  |  |  |  |  |  |  |  |  | X |
| *Patient – DISCUS* |  |  |  |  |  |  |  |  |  | X |
| *Patient – ISMI* |  |  |  |  |  |  |  |  |  | X |
| *Patient – BACE* |  |  |  |  |  |  |  |  |  | X |
| *Patient – CSRI* |  |  |  |  |  |  |  |  | X | X |
| *Qualitative interviews* |  |  |  |  |  |  |  |  |  | X |
| Psychiatrist Administered |  | | | | | | | | | |
| *Patient – SCID-5-RV* |  |  |  |  |  |  |  |  | X |  |

**Abbreviations**: SDS, Social Distance Scale; IAT, Implicit Association Test; ENACT, Enhancing Assessment of Common Therapeutic Factors; WHODAS, World Health Organization Disability Assessment Schedule; PHQ9, Patient Health Questionnaire; GAD7, Generalized Anxiety Disorder; PANSS, Positive and Negative Syndrome Scale; EQ-5D-5L, EuroQol 5 Dimension quality of life scale; BACE, Barriers to Access to Care Evaluation; CSRI, Client Service Receipt Inventory; SCID-5-RV, Structured Clinical Interview for DSM-5-Research Version; ASEC, Antidepressant Side Effect Checklist; AIMS, Abnormal Involuntary Movement Scale.

**Concealment mechanism and implementation**

A biostatistician based at the Duke Global Health Institute and who is not involved in the study will generate the allocation sequence for the 24 municipalities randomized in the study (i.e., concealment applies to this biostatistician). After randomization, the district study coordinator and clinical supervisor in Nepal will be informed because they will need to make the logistical arrangements with PWLE for the PhotoVoice training and RESHAPE-based training. The district study coordinator in collaboration with municipal health coordinators will enroll PCPs for participation in the training. Research assistants will enroll patients.

**Assignment of interventions: masking and procedure for unmasking**

Regarding masking for Objective 1, PCPs will be told that different training approaches are being compared, but they will not be told the condition that is different (i.e., participation of PWLE and aspirational figures). [*Of note, we have used the term ‘*masking’* rather than ‘blinding’ throughout this section based on concerns of the stigmatizing implications of the term ‘blinding’.] The mental health specialist trainers will also be told that different training approaches are being used, but will not be told specific details of the different implementation approaches. Questions will used at key junctures of the study to monitor unmasking: “What was this study evaluating?” If the participant or study staff says something to the effect of: “The study is comparing different training models, then probe to ask about what was different in the training models. If they say involvement of PWLE, then the following questions will be asked: “when did you know about this difference?”, “how did you find about this difference?”, and “which patients, health workers or others are in which study arm?”

For Objective 2, patients will be told that PCPs have received different training strategies but that the intervention they are receiving is the same content for them and surrounding municipalities. Data collectors will be masked throughout collection and study statisticians will be masked during the analysis phase. These are different statisticians from those involved in the randomization. Because training data collection happens on Day 1 and Day 7 of the training when no PWLE are present, this reduces the risk of unmasking to research team members involved in outcome data collection. Because there are no differences in the treatment in each arm, we do not anticipate unmasking patients.

**Collection of outcomes** (additional details)

The qualitative studies are conducted after completion of all of the quantitative collection because qualitative questions have high potential for unmasking research assistants and PCPs, e.g., questions about “what did you think of PWLE participating in your training”, would only be asked in the RESHAPE arm. To promote retention and follow-up, multiple forms of contact information will be collected for patients and their caregivers. Patients will receive compensation, similar to that mentioned above, for all follow-up interviews.

**Criteria for discontinuing or modifying allocated interventions, adherence to interventions, concomitant care, and post-trial care**

Because the mhGAP-IG recommended patient treatments are the same in both arms, there are no criteria for discontinuing one arm. Given that this is an implementation trial, we will not plan formal stopping rules based on a comparison between arms and therefore we will not plan interim analyses of effectiveness. Our primary outcome is detection, not effectiveness of care, given that treatment is expected to be comparable across arms. Because we have SCID interviews at 3-months post patient-enrollment which will determine levels of missed detection or inaccurate detection, stopping rules would not add additional safety for patients. It is not feasible to conduct SCID interviews for all patients in a facility at the time of screening, which would also be a significant deviation from current standard of care. There are no additional strategies as part of the research efforts to improve adherence to mhGAP-IG recommendations. Instead, evaluating adherence will be one of the secondary implementation outcomes. No care provided in primary care facilities nor specialty mental health service is prohibited for this trial. Counseling and psychosocial services are permitted and will be documented. Government health services will continue to be available after the trial is concluded. The DSMB will oversee any modifications or stopping of the trial.

**Eligibility criteria** (additional details)

*Primary care providers (PCPs):* All PCPs with prescribing privileges from the facilities included in the study will be invited to participate. They have undergone training to prescribe non-psychiatric medications in the general training. PCPs will need to have permission from their health supervisor to attend the entire duration of the training, which will be arranged for all health workers in advance of the training. To assure that health facilities do not go unstaffed, we will divide the health trainings into two batches of prescribers, grouped with other health facilities in the municipality, and nearby municipalities in the same implementation arm.

*Patients*: Patients with non-emergency medical needs presenting to the primary care facilities will be randomly selected to participate. The inclusion age range will be 16 years or older, with no upper age limit. We will include participants aged 16 and 17 years, in addition to adult populations. The rationale for including this age group is that the majority of first suicide attempts among women are at this age. In rural Nepal, 90% of completed suicides among women occur before the age of 25 [10], and the majority of first attempts are in the mid to late teens [11]. If the study only included ages 18 and above, the opportunity would be missed to determine if health workers were accurately identifying youth at risk of suicide and making diagnostic and treatment recommendations when appropriate. Within the Nepal mhGAP, there are modules for suicidality/self-harm and child and adolescent mental health needs. Therefore, this is within the scope of the PCPs’ training and the supervision they will receive. In rural areas, such as where this study is being conducted, 16- and 17-year-old youth often assume adult roles and have expectations as adults in the community. The proposed tools have previously been used in 16- and 17-year-old participants and we can therefore collect the same outcome measures. The treatment regimen will also be comparable for this age group.

**Description of consent/assent procedures**

Informed consent will be obtained from all participants prior to inclusion into the study. Informed consent forms will be written to be easily understood by lay persons, enabling them to understand the aims, procedures and potential risks of participation. All consent processes will be conducted by trained research personnel fluent in the relevant local languages. All PCPs in selected health facilities will be recruited for the trainings. They will be recruited based on the randomization of their health clusters into the training as usual versus training plus anti-stigma component. We will obtain written consent from the PCPs for the evaluations when they come for the training. Because of legal requirements in Nepal, for patients who are 16 or 17 years of age, we will seek assent together with signed caregiver permission from one parent or another legal guardian.

It is anticipated that all patients will be able to speak Nepali and will complete the research interview with the research assistants, i.e., interviewer administered. If patients are unable to speak Nepali, research assistants (who are hired from the local communities) will document when an interview is conducted in a different language. Patients meeting criteria for the mental illnesses in the mhGAP list who are unable to demonstrate verbal comprehension (e.g., secondary to a psychotic condition, selective mutism, or other psychiatric reason) will have their caregiver be given the opportunity to consent for participation. Caregivers will only consent for patients if they are their legal guardian. Patients needing acute psychiatric services (e.g., presenting with suicide attempts, alcohol withdrawal, psychosis/mania that cannot be managed in a community setting) will be referred for immediate evaluation by a mental health professional. A protocol will be developed for people in crisis. Because these patients will typically be followed-up at the primary care center after hospitalization, they can be recruited at a later time if eligible. There will be a protocol for researchers and patients in case of mental health deterioration.

**Confidentiality**

Disclosure of medical or other personal information, particularly related to mental health, may pose personal or social risks, especially within community and family settings. The following precautions will promote the privacy of participants and maintain confidentiality of research data: (1) All study staff will be well trained and will receive ongoing supervision by research coordinators in confidentiality and data security procedures, specifically in ethical conduct, confidentiality protection, mandated reporting, and other topics of human participant protection. In addition, the importance of confidentiality will be emphasized with clinical staff (other than PCPs) who may have knowledge about patients’ eligibility or participation in the study. (2) As part of the consent procedure, participants will be informed of the limits of confidentiality (harm to self or others) and mandated reporting requirements. (3) Privacy will be maintained by conducting all interviews, discussions, study assessments, and intervention sessions in closed and private clinic rooms, or at TPO Nepal offices or at home, based on client’s discretion and need. (4) Each participant will be assigned a unique study ID number; direct identifiers will be excluded from the analytic data files before analysis. (5) For minors participating in the study (16-17-year-olds), the minors and their consenting guardians will be informed during the consent procedure that information provided by the minor to the study team is confidential and will not be shared outside the study team. The exceptions to this include imminent risk of harm to self or others, acute medical needs requiring treatment, and violation of national laws related to child protection.

**Oversight and monitoring: Data and Safety Monitoring Board (DSMB), reporting of adverse events, auditing trial conduct, and reporting modifications**

The DSMB, which is comprised of experts in cRCTs, Nepal mental health, and global mental trials, will meet on a twice-yearly basis after initiation of data collection. The DSMB will be responsible for monitoring recruitment progress, adverse events, serious adverse events, deaths related to study participation, and protocol violations. Although the study does not contain *a priori* stopping rules, the DSMB can review progress after each of the first two (of three) waves to recommend any modifications needed to study procedures to optimize safety of participants. During research assistants’ interactions with participants, patients will be referred for any emergency psychiatric needs that have not been addressed by the PCP, with a specific procedure for acute suicidality.

Data auditing procedures, via sampling from the quantitative and qualitative data collected, will be undertaken to assure the viability and integrity of the data prior to analysis. Research log books will be completed by all research workers to establish full details of the collection of original primary data sources. All interviews will be conducted by trained research personnel fluent in Nepali. Regular site audits will be completed by the Nepal Health Research Council. Protocol amendments will be made in ClinicalTrials.gov, and modifications will be reported to George Washington University institutional review board and the Nepal Health Research Council.

**Sample size** (additional details)

For Objective 1 comparing 6-month post-training PCP stigma (measured using the social distance scale [SDS]), if 3 PCPs per health facility (HF) are followed-up at 6-months we estimated 80% power to detect a standardized effect size of 0.42 for the comparison of the RESHAPE implementation strategy vs. IAU, or a standardized effect size of 0.51 if only 2 PCPs/HF provide data. This was based on the following assumptions: 24 municipalities (12 per arm), 3 health facilities per municipality and 3 PCPs per health facility enrolled in the study; pairwise correlation of SDS of 0.02 and 0.01 for pairs of PCPs in the same health facility and for pairs of PCPs in different health facilities but within the same municipality, respectively, which are assumed the same in both implementation arms. This calculation was performed at the 5% significance level using equation (4) of Wang et al. [13] with variance of the RESHAPE effect ($\sigma_{\beta}^{2}$) estimated using the term to the right of the “x” symbol in equation (8) of Wang et al. [13]. In practice, we anticipate that there will be, on average, 2.4 PCPs/HF followed-up at 6-months post-training so that we would be able to detect effect sizes somewhere between 0.42-0.51 with 80% power. Standardized effect sizes of 0.49 for 3 PCPs/HF and 0.59 for 2 PCPs/HF would be detectable with 90% power if all other assumptions hold.

For Objective 2, we estimated 97% power to detect a ratio of 1.40 comparing the proportion of PCP diagnoses that are accurate (as determined by psychiatrist evaluation using SCID at 3-months) in RESHAPE vs. IAU arms. This ratio is based on comparing 69.9% accurate diagnoses in RESHAPE to 50.1% accurate diagnoses in IAU. See Figure S3 for derivation of these proportions, including key assumptions including the proportion screened positive (i.e., diagnosed positive by PCP), where we note that the assumed proportions diagnosed positive by PCP are small based on pilot data and conservative assumptions (only 2% in IAU which is based on pilot data collected by our team, and 3% in RESHAPE based on a very conservative assumption in the increase in the proportion with a diagnosis). Additional assumptions are: 24 municipalities (12 per arm), 3 health facilities per municipality, 1 PCP per health facility providing MH services per health facility and 16 patients per PCP evaluated for accuracy of diagnosis at 3 months; pairwise correlation of accurate diagnosis of a patient is 0.05 for pairs of patients of the same PCP (equivalently of the same health facility since we assume 1 PCP provides MH services per health facility), and 0.03 for pairs of patients of different PCPs in different health facilities but in the same municipality, respectively and are assumed the same in both implementation arms. This calculation was performed at the 5% significance level using equation (4) of Wang et al. [13] with variance estimated using the term to the right of the “x” symbol in equation (11) of Wang et al. [13], which assumes a log link (and therefore assumes b = log(0.699) – log(0.501) = 0.333).

Importantly, this comparison is applicable to patients for whom a psychiatrist makes a (gold-standard) diagnosis at the 3-month follow-up. Because our study can only recruit a sub-sample of those who do not receive a PCP diagnosis (estimated to be 40% in both arms, see sampling fraction negative [sfn]=40% in Fig. S3) and 3-month follow-up can only include sub-samples of recruited patients who did not receive a diagnosis from a PCP (expected to be 10% [sfnn] of those who are true negatives and 50% [sfnp] of those who are false negatives in both arms), the between-arm comparison applies to a population which, compared to the general health facility-visiting population, has an overrepresentation of those who screen positive (i.e. are PCP-positive).

**Figure S3**. Key assumed proportions for Objective 2 implementation power calculation at sample level.

Note: this figure shows how the final sample is expected to show 50.1% IAU vs. 69.9% RESHAPE with accurate diagnosis. Note: sf= sampling fraction; sfn = sampling fraction of PCP-diagnosed negative is assumed to be 40% in both arms; sfnn= sampling fraction of PCP-diagnosed negatives and psychiatrist negatives (i.e., true negatives) is assumed to be 10% in both arms; sfnp= sampling fraction of PCP-diagnosed negatives and psychiatrist positives (i.e., false negatives) is assumed to be 50% in both arms.

Sensitivity to assumptions for the power calculation of the accuracy outcome is shown in **Table S2**, whereby 2 PCPs/HF deliver MH services rather than 1 PCP/HF. In this case, power is expected to increase slightly when the pairwise correlation of accuracy of diagnosis of patients of two different providers in the same health facility (0.04) is assumed to lie between that for patients of the same provider (0.05) or for patients of different providers in the same municipality (0.03). Similarly, we expect no decline in power in the case that the assumed fraction of patients with a PCP diagnosis of mental illness is slightly different to that assumed above (namely, 2% in IAU and 3% in RESHAPE).

| **Table S2: Sensitivity of power to number of primary care providers (PCP) per health facility (HF) - sample size assumptions** | | | | |
| --- | --- | --- | --- | --- |
| Sample size assumption/parameter | IAU | RESHAPE | | Total (across both arms) |
|  |  |  | |  |
| Aim 2 |  |  | |  |
| PCP/HF providing MH services to patients | 1 | 1 | | 72 |
| Patients/HF^a^ enrolled | 80 | 80 | | 5760 |
| Patients with SCID^b^ at 3-mth FU | 16 | 16 | | 1152 |
| Accuracy pairwise correlation |  |  | |  |
| For patient of same PCP | 0.05 | 0.05 | | 0.05 |
| For patient of different PCP in same municipality^c^ | 0.03 | 0.03 | | 0.03 |
| Assumed diagnostic accuracy^d^ | 50.1% | 69.9% | | - |
| Effect size | Ratio = 1.40; Diff = 19.8% | | | - |
| Power | 97% | | | - |
|  |  | | |  |
|  |  | | |  |
| PCP/HF providing MH services to patients | 2 | | 2 | 144 |
| Patients/PCP enrolled | 40 | | 40 | 5760 |
| Patients/PCP with SCID^b^ at 3-mth FU | 8 | | 8 | 1152 |
| Accuracy pairwise correlation |  | | |  |
| For patient of same PCP | 0.05 | | 0.05 | 0.05 |
| For patient of different PCP in same HF | 0.04 | | 0.04 | 0.04 |
| For patient of different PCP in same municipality | 0.03 | | 0.03 | 0.03 |
| Assumed diagnostic accuracy^d^ | 50.1% | | 69.9% | - |
| Effect size | Ratio = 1.40; Diff = 19.8% | | | - |
| Power | 97% | | | - |
|  |  | | |  |
| ^a^ Equivalently Patients/PCP given assumption of 1 PCP/HF providing MH services, and note that it is assumed that all PCP-positive patients are enrolled in the study whereas only a sub-sample of PCP-negative patients are enrolled (see assumed sampling fractions in Figure S3); ^b^ Based on assumed sub-sampling of PCP-negatives at 3-month follow-up so that only a sub-sample of enrolled PCP-negative patients are assessed for diagnostic confirmation (by psychiatrist using the SCID) and that a percentage are lost to follow-up (see assumptions in Figure S3); ^c^ Assuming 1 PCP per HF provides MH services (note, if multiple PCP per HF, an additional level of clustering would be added); ^d^ For calculation, see Figure S3. | | | | |

| **Table S3: Sensitivity of power to proportion of patients identified as PCP-positive** | | | | | |
| --- | --- | --- | --- | --- | --- |
| **IAU** | **RESHAPE** | **Difference** | **Total # of patients enrolled** | **Total # of patients at 3-mth FU** | **Power** |
| 2% | 3% | +1% | 5760 | 1152 | 97% |
| 1% | 2% | +1% | 5760 | 1080 | 98% |
| 2% | 4% | +2% | 5760 | 1224 | 98% |
| 1% | 3% | +2% | 5760 | 1152 | 99% |
|  | | | | | |

**Statistical methods** (additional details)

Additionally, for Objective 1, pre-training SDS will be included as a covariate to leverage the information in each primary care worker’s baseline level and, because there are three SDS follow-up measurements (post-training, 3- and 6-months follow-up), these will be simultaneously modeled. As such, time point and the implementation-arm by time interaction will be included in the stigma model in order to estimate different effects at different follow-up time points, with 6-months the primary time point. Both outcomes are specified within a four-level structure: municipality, health facility, PCP and time point for stigma; municipality, health facility, PCP and patient for diagnostic accuracy. In practice, we note that, for the latter, because we expect 1 PCP per health facility who provides MH services, the PCP and health facility will coincide thus reducing the structure to three levels. Moreover, we expect to collapse to a PCP measure of diagnostic accuracy, analyzed within a binomial model (with a denominator of accurate diagnoses and numerator of all diagnoses made) for computational efficiency and because we do not expect primary analyses to include patient-level characteristics. For objective 1, the identity link will be used to estimate between-arm differences in mean PCP stigma (SDS) whereas the log and identity links will be used to estimate the ratio and difference, respectively, in the proportion of patients with accurate diagnosis. We expect to use the nested exchangeable correlation when a three level structure is analyzed [12] and the extended nested exchangeable correlation structure when a four-level structure is analyzed [13] e.g., in the situation whereby there is more than 1 PCP per health facility who provides MH services.

There are two important distinctions between the modeling approach for the two outcomes. First, related to the sampling scheme, and, second, in the nature of missing data. Regarding the sampling scheme, all suspected ‘positive’ patients will be interviewed by a psychiatrist at 3-months post-baseline whereas only a sub-sample of suspected ‘negative’ patients will be interviewed. Therefore, in practice, accuracy will only be available for patients who were interviewed three months after enrollment. Secondary analysis will account for our sampling design by using sampling weights, whereas our primary analysis will not. That is, our primary analysis will assess the impact of the RESHAPE implementation strategy on the accuracy outcome for a population of patients that is comparable to those included in the sample of patients at 3-month follow-up. This is aligned with the research question for which we powered objective 2. If there are model convergence issues for the accuracy outcome models, a modified Poisson approach [14] will be used or, instead, odds ratios will be estimated using a logistic approach. All analyses will be reported in accordance with recommendations laid out in the CONSORT statement on the reporting of cRCTs [15].

**Methods in analysis to handle protocol non-adherence and any statistical methods to handle missing data**

Fidelity to the RESHAPE implementation will be assessed, summarized and results as to the effectiveness of the RESHAPE implementation strategy will be interpreted in light of the degree of adherence to the implementation strategy. Missing longitudinal data could arise for either PCPs or for the patients who are followed over time. For PCPs, our previous studies have demonstrated minimal drop-out (89% participation at 4-months post-training) [16]. For patients, by design, only a sub-sample will be followed over time (specifically, to three months, only screen-positive and a sub-sample of screen-negatives and to six months, only those with a “true positive” diagnosis by a psychiatrist at three months). To address missing longitudinal outcomes, we will adopt two strategies, which we explain here using the six-month PCP SDS outcome as an example. First, we will identify pre-training variables predictive of missing outcome (i.e., a covariate-dependent missing-at-random pattern) by summarizing baseline covariates stratified by individuals missing SDS and those not missing SDS. Then, in a sensitivity analysis, we will include those as predictors in the main analysis model. Second, we will perform additional sensitivity analyses using a pattern-mixture approach that tests sensitivity to the missing-at-random assumption by positing a range of missing-not-at random patterns [17]. As such, through these comprehensive analyses, we will seek to demonstrate whether there is evidence to suggest that the estimated effect of the RESHAPE implementation strategy is robust to our assumptions.

**Interim analyses**

No formal interim analyses are currently planned given that the treatments received by patients are the same in both implementation arms.

**Methods for additional analyses**

Heterogeneity analyses are pre-specified by gender for both primary outcomes (PCP stigma and patient accurate diagnosis). This is because there may be gender differences in stigma and in clinical practices. In particular, an interaction between gender and implementation arm will be included in models. For secondary outcomes for both primary care workers and for patients, analytic methods are comparable to those described for the two primary outcomes. Moreover, for PCP outcomes with repeated measurements, secondary time points of interest (including the SDS primary outcome measure) will be immediately post-training and 3-months post-training, in addition to the six-month primary time point of interest.

Cost-effectiveness will be assessed from the primary care/health sector perspective. The analysis will examine the arm differences in Quality-of-Life Years (QALYs) measured with the EQ-5D-5L compared to arm differences in health system and patient costs (see **Supplemental Figure 4**). In keeping with the Type 3 Hybrid Implementation-Effectiveness approach, we will conduct two cost-effectiveness analyses. The first analysis will be a standard cost-effectiveness of treatment. This will be limited to exploring the cost differences in the two implementation arms focusing only on the patients evaluated at 6-months (i.e., the “true positives”). This analysis is limited to the question of “among those patients who are correctly diagnosed, what is the cost difference per quality adjusted life year gained.” Given the non-inferiority nature of this trial at the “true positive” patient level, we do not anticipate to demonstrate cost-effectiveness of RESHAPE over IAU when limiting to only those correctly diagnosed and treated. This is because, accurate treatment was above 90% in standard Nepal implementation of mhGAP in prior studies. Therefore, we will additionally conduct a cost-effectiveness of implementation strategy analysis. This second analysis takes into account the total distribution of true positive, true negative, false positive, and false negative patients based on the diagnostic accuracy study. For this analysis, we hypothesize cost-effectiveness because the total number of correctly categorized and treated patients will be greater in the RESHAPE implementation strategy. Thus, although at the level of individual patients diagnosed correctly, RESHAPE will not be more cost-effective, at the total primary care patient population level, the RESHAPE will be cost effective because the *reach* of accurate treatment is greater and the wastage of resources on unnecessary treatments, which would be measured in cost of care both at the primary care level but also any other services such as sought specialty care or hospitalization, is lower. The cost-effectiveness of treatment will take an economic evaluation alongside trial approach, and the cost-effectiveness of implementation strategy will take a decision modelling approach. Results on utilities and costs from the patient outcomes in the treatment analysis will be used to parameterize the decision model, i.e., the cost-effectiveness of treatment analysis is necessary to conduct the decision modeling for the implementation strategy analysis.


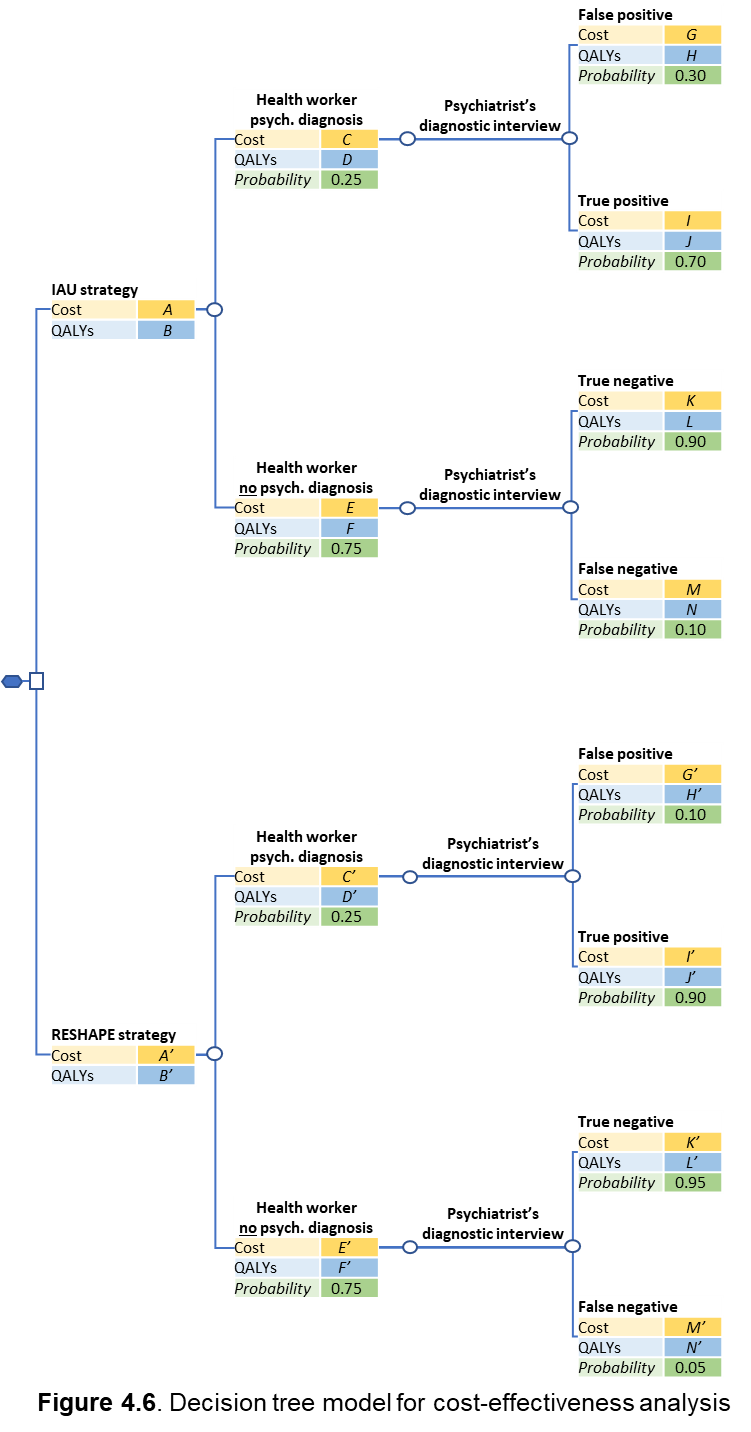


**Figure S4**. Decision tree model for cost-effectiveness analysis.

**Access to the full protocol, participant level-data and statistical code**

The protocol is registered through ClinicalTrials.gov. Participant level-data will be available through NIMH Data Archive. The statistical code will be made available at the time of publication.

**Data management**

RESHAPE will be collecting primary data in a variety of different ways, including: electronic data collection applications, paper forms, and video- and audio- recording devices. We will also obtain secondary data from health facilities. For electronic data collection, the following three applications will be used: Research Electronic Data Capture (REDCap), the WHO Ensuring Quality in Psychological Support (EQUIP) platform, and a version of the Implicit Association Test (IAT). REDCap is a secure, web-based application designed to support data capture. RESHAPE uses the Duke School of Medicine instance of REDCap, which is managed and supported by the Duke Office of Clinical Research. To accommodate offline data collection, we use the REDCap Mobile application. It integrates with REDCap and is also supported and maintained by Duke University. REDCap stores data in a database and mediates access, which preserves original data values and prevents overwriting, deletions, or other deprecations of raw data integrity. REDCap’s robust data quality protection features include: audit trails, tiered access permissions, data validation (format and range restrictions, skip logic, etc.), and restriction of any modifications to instruments and data after collection begins. Structured role plays to collect competency data on PCPs will be collected in the EQUIP platform which includes the ENACT tool in Nepali and an mhGAP competency assessment tool, also in Nepali [18]. A version of IAT was developed specifically for administration in Nepali using touch screens on tablets. Health facility data will be collected by outpatient registers. Paper forms will be kept in a locked cabinet in a locked room of a locked office operated by TPO Nepal. Audio and video files will be stored on GW Box.

**Dissemination plans**

We plan to engage the federal, provincial, and local governments, as well as communities. The study findings and intervention processes will be disseminated in scientific communities through articles published in national or international peer-reviewed journals in collaboration with PWLE. The findings will also be presented in professional conferences, seminars, workshops, and meetings. RESHAPE materials will be made freely available to anyone implementing mental health training of PCPs. Feedback will also be provided to PWLE and PCPs.

**References**

1. Kohrt BA, Luitel NP, Acharya P, Jordans MJD: **Detection of depression in low resource settings: validation of the Patient Health Questionnaire (PHQ-9) and cultural concepts of distress in Nepal**. *BMC Psychiatry* 2016, **16**(1):e58.

2. Jordans MJD, Aldridge L, Luitel NP, Baingana F, Kohrt BA: **Evaluation of outcomes for psychosis and epilepsy treatment delivered by primary health care workers in Nepal: a cohort study**. *International Journal of Mental Health Systems* 2017, **11**(1):70.

3. Pradhan B, Chappuis F, Baral D, Karki P, Rijal S, Hadengue A, Gache P: **The alcohol use disorders identification test (AUDIT): validation of a Nepali version for the detection of alcohol use disorders and hazardous drinking in medical settings**. *Substance Abuse Treatment, Prevention, and Policy* 2012, **7**(1):42.

4. World Health Organization: **World Health Organization disabilty assessment schedule: WHODAS II. Phase 2 field trials. Health services research**. 2000.

5. Pattanaphesaj J, Thavorncharoensap M, Ramos-Goñi JM, Tongsiri S, Ingsrisawang L, Teerawattananon Y: **The EQ-5D-5L Valuation study in Thailand**. *Expert review of pharmacoeconomics & outcomes research* 2018, **18**(5):551-558.

6. De Silva MJ, Rathod SD, Hanlon C, Breuer E, Chisholm D, Fekadu A, Jordans M, Kigozi F, Petersen I, Shidhaye R *et al*: **Evaluation of district mental healthcare plans: the PRIME consortium methodology**. *Br J Psychiatry* 2016, **208 Suppl 56**:s63-70.

7. Luitel NP, Jordans MJD, Kohrt BA, Rathod SD, Komproe IH: **Treatment gap and barriers for mental health care: A cross-sectional community survey in Nepal**. *PLOS ONE* 2017, **12**(8):e0183223.

8. Brohan E, Clement S, Rose D, Sartorius N, Slade M, Thornicroft G: **Development and psychometric evaluation of the Discrimination and Stigma Scale (DISC)**. *Psychiatry Research* 2013, **208**(1):33-40.

9. Ritsher JB, Otilingam PG, Grajales M: **Internalized stigma of mental illness: psychometric properties of a new measure**. *Psychiatry research* 2003, **121**(1):31-49.

10. Hagaman AK, Khadka S, Lohani S, Kohrt B: **Suicide in Nepal: a modified psychological autopsy investigation from randomly selected police cases between 2013 and 2015**. *Social Psychiatry and Psychiatric Epidemiology* 2017, **52**(12):1483-1494.

11. Kohrt BA, Worthman CM, Ressler KJ, Mercer KB, Upadhaya N, Koirala S, Nepal MK, Sharma VD, Binder EB: **Cross-cultural gene−environment interactions in depression, post-traumatic stress disorder, and the cortisol awakening response: FKBP5 polymorphisms and childhood trauma in South Asia**. *International Review of Psychiatry* 2015, **27**(3):180-196.

12. Teerenstra S, Lu B, Preisser JS, Van Achterberg T, Borm GF: **Sample size considerations for GEE analyses of three‐level cluster randomized trials**. *Biometrics* 2010, **66**(4):1230-1237.

13. Wang X, Turner EL, Preisser JS, Li F. **Power considerations for generalized estimating equations analyses of four-level cluster randomized trials**. *Biom J*. 2022 Apr;64(4):663-680. doi: 10.1002/bimj.202100081.

14. Zou GY, Donner A: **Extension of the modified Poisson regression model to prospective studies with correlated binary data**. *Stat Methods Med Res* 2013, **22**(6):661-670.

15. Campbell MK, Piaggio G, Elbourne DR, Altman DG: **Consort 2010 statement: extension to cluster randomised trials**. *BMJ: British Medical Journal* 2012, **345**.

16. Kohrt BA, Jordans MJD, Turner EL, Rai S, Gurung D, Dhakal M, Bhardwaj A, Lamichhane J, Singla DR, Lund C *et al*: **Collaboration with people with lived experience of mental illness to reduce stigma and improve primary care services in Nepal: a pilot cluster randomized clinical trial**. *JAMA Network Open* 2021, **4**(11):e2131475.

17. Fiero MH, Hsu CH, Bell ML: **A pattern-mixture model approach for handling missing continuous outcome data in longitudinal cluster randomized trials**. *Stat Med* 2017, **36**(26):4094-4105.

18. Kohrt BA, Schafer A, Willhoite A, van't Hof E, Pedersen GA, Watts S, Ottman K, Carswell K, van Ommeren M: **Ensuring Quality in Psychological Support (WHO EQUIP): developing a competent global workforce**. *World Psychiatry* 2020, **19**(1):115-116.
